# Deep Eutectic Inks for Multiphoton 3D Laser Microprinting

**DOI:** 10.1002/adma.202507640

**Published:** 2025-06-25

**Authors:** Philipp Mainik, Christoph A. Spiegel, Jonathan L.G. Schneider, Martin Wegener, Eva Blasco

**Affiliations:** ^1^ Institute for Molecular Systems Engineering and Advanced Materials (IMSEAM) Heidelberg University 69120 Heidelberg Germany; ^2^ Institute of Organic Chemistry (OCI) Heidelberg University 69120 Heidelberg Germany; ^3^ Institute of Applied Physics Karlsruhe Institute of Technology (KIT) 76131 Karlsruhe Germany; ^4^ Institute of Nanotechnology Karlsruhe Institute of Technology (KIT) 76344 Eggenstein‐Leopoldshafen Germany

**Keywords:** 4D printing, light‐based 3D printing, polymerizable deep eutectic solvents, soft materials, stimuli‐responsive polymers

## Abstract

Multiphoton 3D laser printing of polymers has become a widespread technology for manufacturing 3D architectures on the micro‐ and nanometer scale, with booming applications in micro‐optics, micro‐robotics, and micro‐scaffolds for biological cell culture. However, many applications demand material properties that are not accessible by conventional polymer inks. These include large stiffness, for which recent breakthroughs based on inorganic materials have been reported. Conversely, some applications require very low stiffness and high mechanical compliance. Existing solutions achieve softness by low crosslinking densities, at the inherent expense of deteriorated spatial resolution and structure quality. Herein, this apparent contradiction is resolved by introducing multiphoton inks based on deep eutectic systems, comprising Lewis or Brønsted acids/bases. The 3D printed materials support extremely large strains and bulk Young's moduli as low as 260 kPa under aqueous conditions, well suited for biological applications – at comparable ease of use and spatial resolution as well‐established commercially available polymer inks.

## Graphical abstract

Using deep eutectic inks in multiphoton 3D laser printing enables excellent printability of soft and elastic complex 3D microstructures. This is achieved by employing supramolecular interactions between a variety of monomers and a metal halide, which is easily removed during development. The new ink design is extendable to other monomer compositions to achieve multifunctional and stimuli‐responsive 4D microprinted materials.

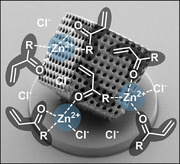

## 1 Introduction

3D microfabrication by multiphoton 3D laser printing (MPLP), also known as multiphoton lithography or direct laser writing, offers unparalleled precision and versatility in 3D microfabrication, enabling the creation of complex structures with nanoscale resolution.^[^ ^1^, ^2^, ^3^, ^4^, ^5^, ^6^ ^]^ This technology is driving advancements across a wide range of cutting‐edge applications, from optics and photonics^[^ ^7^, ^8^, ^9^, ^10^, ^11^, ^12^, ^13^ ^]^ to soft‐robotics,^[^ ^14^, ^15^, ^16^, ^17^, ^18^, ^19^, ^20^ ^]^ and biology.^[^ ^21^, ^22^, ^23^, ^24^ ^]^ As demand for these applications grows, developing printable materials or inks that meet specific requirements is crucial. Recent advances in materials such as fused silica and metal oxides have enabled the fabrication of stiff 3D microstructures,^[^ ^25^, ^26^, ^27^, ^28^, ^29^, ^30^ ^]^ but achieving soft and elastic materials with high precision at these scales remains challenging.

Currently, the design of inks for MPLP typically involves the incorporation of multifunctional monomers, molecules equipped with multiple photopolymerizable moieties, which enable fast efficient polymerization for the generation of stable 3D microstructures.^[^ ^31^, ^32^ ^]^ Thus, good printability is very often inevitably linked to the generation of highly crosslinked polymer networks and stiff materials. Therefore, the design of inks for 3D‐printed soft and elastic structures typically involves an iterative process to find an optimum between desired mechanical properties and printability.^[^ ^5^ ^]^ Reducing the crosslinking density and thereby reducing the stiffness, is usually achieved by either adding monofunctional monomers or solvents which significantly impact the printability by lowering achievable printing speeds and structural quality. Therefore, a major limitation of today's printable materials for MPLP is the lack of functional soft materials that enable fast and precise fabrication.^[^ ^6^, ^33^, ^34^ ^]^ Filling this missing gap with a suitable material approach is of particular interest for advanced (biomedical) applications in the life sciences and flexible electronics.

One approach to overcome this challenge is the use of dynamic bonds, which allow fine‐tuning of the crosslinking density and thus mechanical characteristics after printing. So far, reported strategies usually require multiple steps of chemical synthesis limiting wide‐range applicability.^[^ ^35^, ^36^, ^37^ ^]^ As an alternative, inks using supramolecular interactions based on ionic or hydrogen bonding could offer easily accessible and versatile systems. We have identified deep eutectic systems (DESs) as a promising class of materials for this approach.^[^ ^38^, ^39^ ^]^ DESs are liquid eutectic mixtures comprised of Lewis or Brønsted acids and bases. These mixtures are readily accessible, possess low volatility as well as tunable viscosity, and offer excellent media for dissolving a variety of polar and apolar compounds.^[^ ^38^, ^39^ ^]^ Polymerizable DESs, which contain at least one component that allows polymerization, have very recently raised interest in different fields.^[^ ^40^, ^41^, ^42^, ^43^, ^44^, ^45^, ^46^ ^]^ However, this material class is completely unexplored for the fabrication of functional 3D/4D microstructures using MPLP.

**Figure 1:**
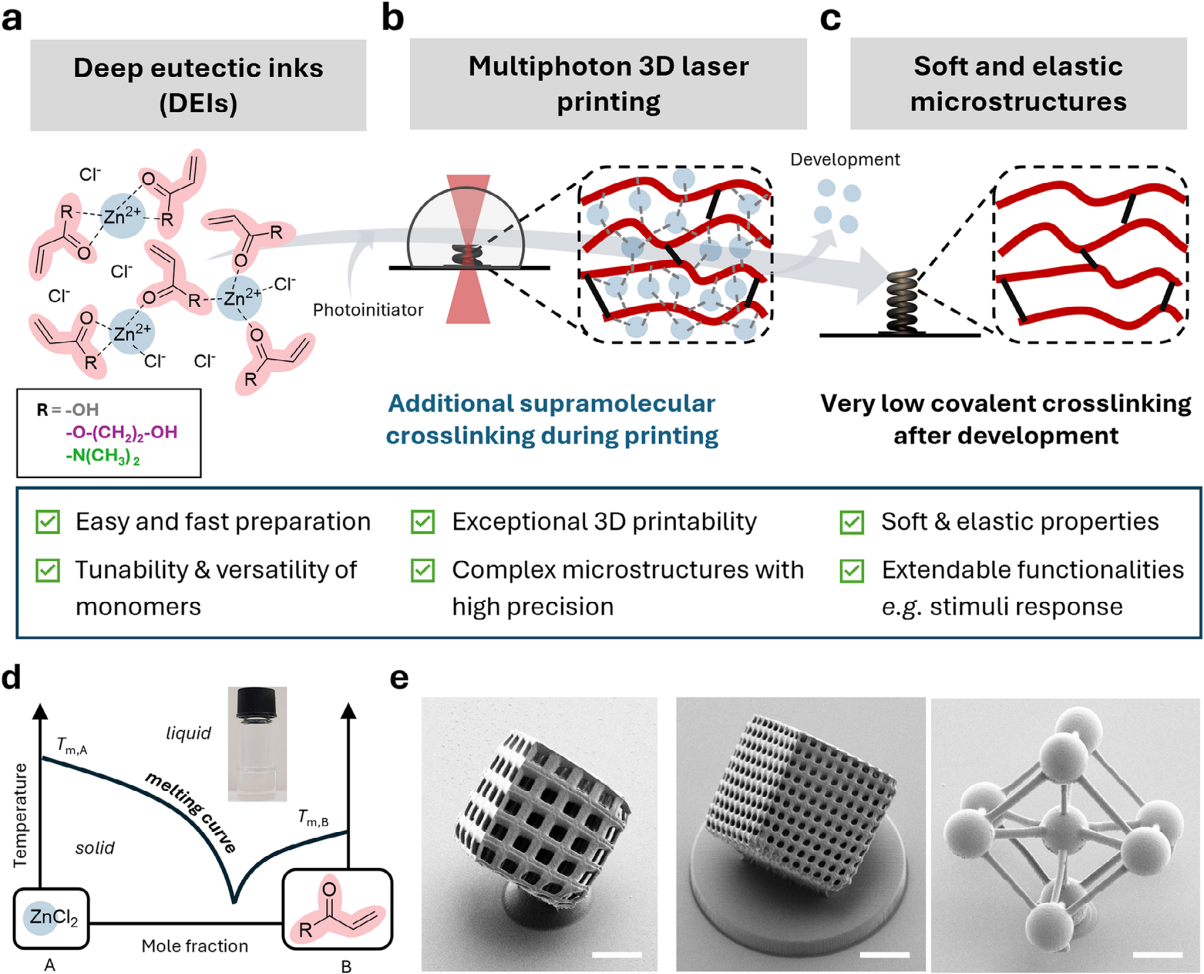
Deep eutectic systems (DESs) as versatile printable materials for multiphoton 3D laser printing (MPLP) enabling complex and soft 3D microstructures. a) We introduce deep eutectic mixtures based on acrylic acid (AAc), 2‐hydroxyethyl acrylate (HEA), and *N*,*N*‐dimethylacrylamide (DMAAm) and zinc chloride (ZnCl_2_). The zinc ions interact with the acrylic monomers through ion‐dipole and hydrogen bonds, forming “multi‐functional” monomers that facilitate rapid photocrosslinking during the printing process. b) The supramolecular crosslinking enables fast printing and enhances the structural stability of the printed objects, allowing for the fabrication of 3D microstructures with intricate geometries and fine features. c) Developing and disrupting the supramolecular ion‐dipole and hydrogen bonds results in a low‐density crosslinked network that leads to highly elastic and soft 3D microstructures. d) Schematic illustration of the DEI phase diagram, where a transparent ink is formed from the 1:2 ZnCl₂/monomer mixtures (shown in the photograph). e) Exemplary scanning electron microscopy (SEM) images of 3D complex microstructures fabricated from DEIs. Scale bars 100 µm.

Herein, we present for the first time the use of DESs for fast and efficient MPLP of soft and flexible 3D structures with high resolution. The printable mixtures consist almost entirely of monofunctional acrylic monomers with zinc chloride (**Figure** **1**). Their unique supramolecular interactions facilitate outstanding printing performances with a wide range of readily available acrylic monomers and only minimal amounts of multifunctional monomers (<5 wt%). This enables rapid fabrication of soft, elastic, and even multi‐responsive structures ranging from µm to mm sizes with high fidelity, that is unattainable by conventional printable materials.

## 2 Results and Discussion

### 2.1 Identification and Characterization of Suitable Deep Eutectic Systems

To bridge the current challenge of efficient MPLP of soft and elastic 3D/4D micro‐ to millimeter‐sized structures, we explored DESs which provide supramolecular bonds during microprinting and can easily break during the development step leading to a low crosslinked network (Figure 1). In particular, deep eutectic mixtures based on a metal halide and photopolymerizable acrylic monomers were explored. Zinc chloride was chosen as a suitable metal halide with a filled d^10^ shell for the preparation of colorless deep eutectic mixtures using different acrylic monomers – acrylic acid (AAc), 2‐hydroxyethyl acrylate (HEA), and *N*,*N*‐dimethylacrylamide (DMAAm) – in a 1:2 ratio ZnCl_2_/monomer. Here, the zinc chloride is expected to form ion‐dipole or hydrogen bonds with the acrylic monomers providing supramolecular entities that act as “multi‐functional” monomers enabling fast crosslinking during MPLP. Afterward, the metal halide can be easily removed leaving behind a slightly crosslinked material.

**Figure 2:**
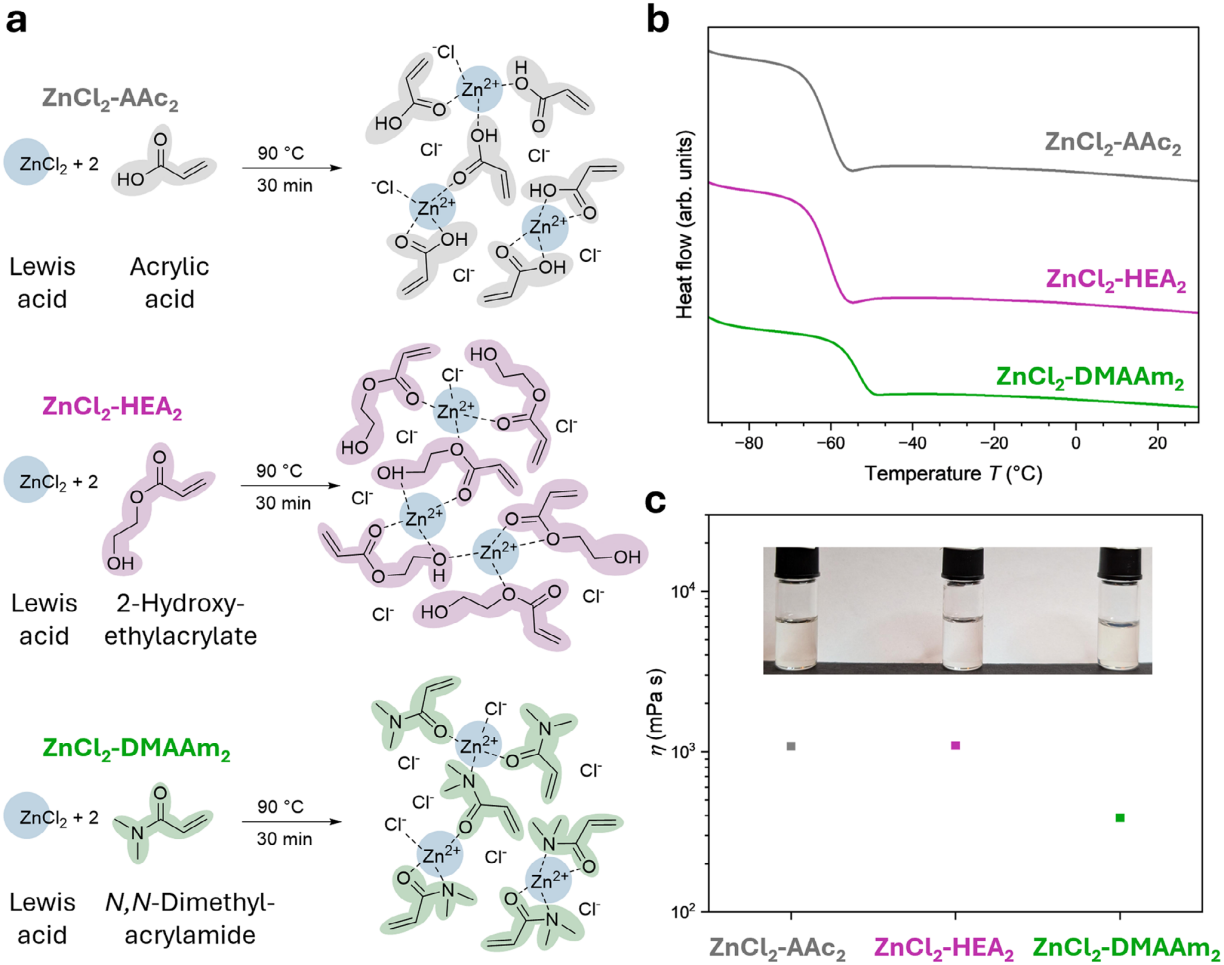
Preparation and characterization of DESs. a) Three different deep eutectic systems (DESs) were easily prepared by mixing acrylic monomers (acrylic acid, 2‐hydroxyethyl acrylate, or *N,N*‐dimethylacrylamide) with zinc chloride as a Lewis acid in a molar ratio of 2:1 at 90 °C for 30 min. b) DSC thermograms of the formed DESs, demonstrating their deep eutectic behavior. The three systems show glass transitions far below room temperature and the absence of additional melting transitions of the acrylic monomers. The thermographs are presented in stack with an offset for clarity. c) Dynamic viscosities of prepared DESs measured by rotational rheology at shear rates of 1000 s^−1^ with photograph of the prepared mixtures showing their optical transparency.

The DESs were prepared in a facile way by stirring both components vigorously at elevated temperatures (90 °C) in a molar ratio *x* of 2:1 acrylic monomer to zinc chloride (see **Figure** **2** and Experimental Section). After 30 min, the zinc chloride and acrylic monomers fully mixed into a colorless liquid which became highly viscous after cooling it to room temperature. The liquid state of the mixtures clearly indicated a phase transition below room temperature and deep eutectic behavior. The observed deep eutectic behavior was confirmed by performing differential scanning calorimetry (DSC) of all the analyzed mixtures (Figure 2). Importantly, the thermograms showed different behavior compared to the single components. For example, the melting point of acrylic acid at *T*_m_ = 9.5 °C was absent in the acrylic acid mixtures, instead a new glass transition appeared at *T*_g_ = −53.8 °C (Figure S1, Supporting Information). Similarly, the two DESs ZnCl_2_‐HEA_2_ and ZnCl_2_‐DMAAm_2_ also exhibited glass transitions at temperatures far below room temperature, i.e., −60.7 and −53.3 °C, respectively. The thermograms align well with previously reported behavior of deep eutectic mixtures.^[^ ^47^, ^48^ ^]^ The preparation of DESs with other molar ratios *x* was also possible allowing precise fine‐tuning of the glass transition (Figure S2, Supporting Information) as well as dynamic viscosity (see Figures S3 and S4, Supporting Information). ^1^H NMR experiments indicated the presence of supramolecular interactions between the zinc chloride and the acrylic monomers (see Supporting Information).

The optical properties of the obtained DESs were also investigated. First, we determined the refractive indices (Figure S12, Supporting Information), which range from *η*_20 °C_ = 1.48–1.52 at the wavelength of the femtosecond pulsed laser *λ* = 780 nm. This range is comparable to indices of various commercially available inks (IP‐S, IP‐Dip, IP‐L, OrmoComp, IP‐Visio, and PO4) frequently used in MPLP.^[^ ^49^ ^]^ Additionally, we have performed UV‐vis and IR spectroscopy to study the absorption of the deep eutectic mixtures (see Supporting Information). Importantly, the three colorless mixtures showed no absorption between *λ* = 380–800 nm making it highly suitable for light‐based laser printing at *λ* = 780 nm (Figure S13, Supporting Information). In addition to that, no photocuring was observed even after exposing them for 5 min to UV irradiation (*λ* = 380–400 nm). The stability under ambient light conditions facilitates future handling of the DESs. FTIR spectroscopy of ZnCl_2_‐AAc_2_, ZnCl_2_‐HEA_2_, and ZnCl_2_‐DMAAm_2_ confirmed the presence of supramolecular interactions between zinc chloride and the employed monomers (Figures S14–S16, Supporting Information).

### 2.2 MPLP of Deep Eutectic Inks

**Figure 3:**
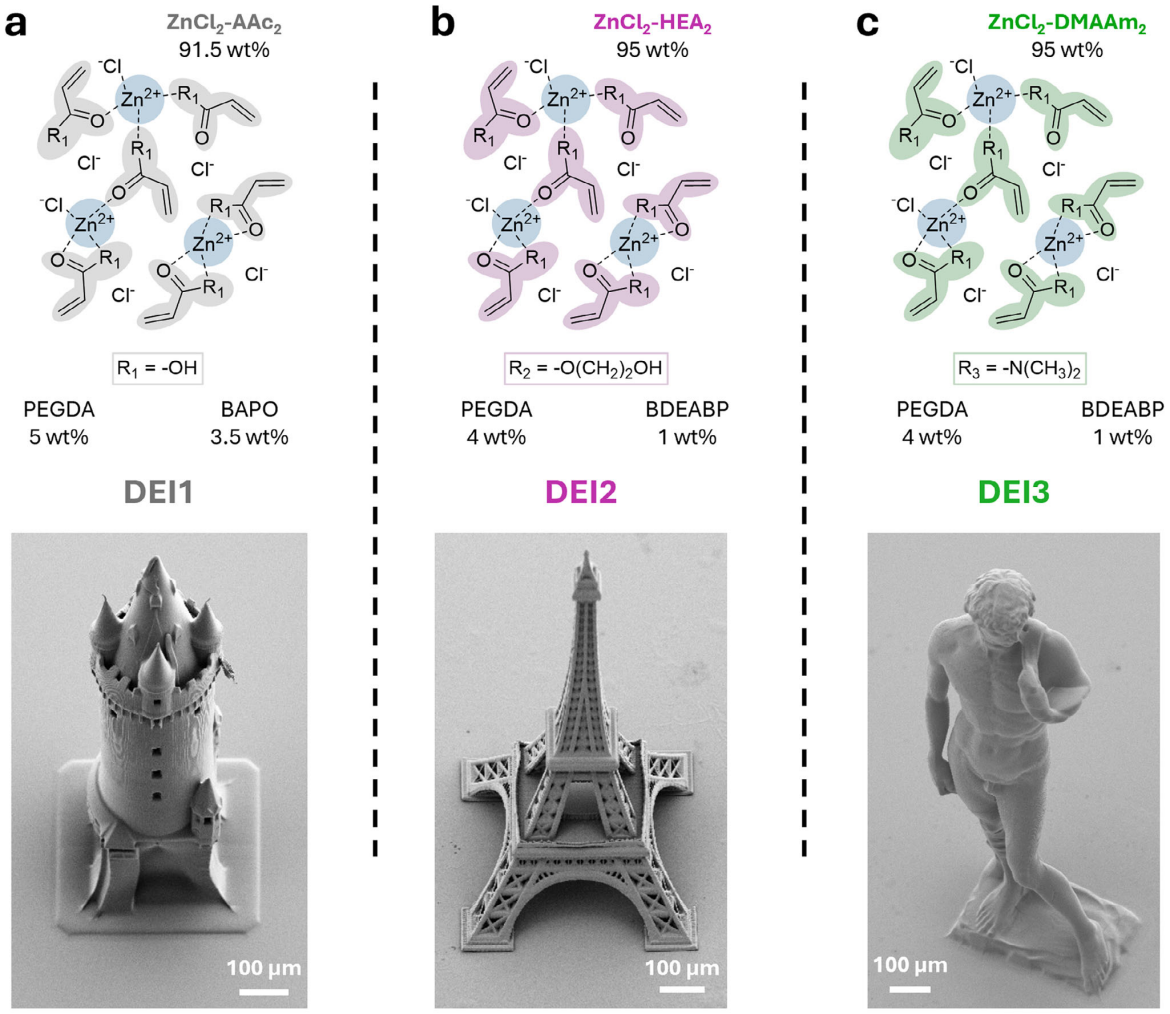
3D microprinting in dip‐in mode MPLP using DEIs. a) Ink composition of DEI1 (top) and exemplary SEM image of DEI1 printed “Eschenheimer Turm” (bottom). b) Ink composition of DEI2 (top) and SEM image of DEI2 printed “Eiffel tower” (bottom). c) Ink composition of DEI3 (top) and SEM image of DEI3 printed “Michelangelo's David”. Notes: The inks have been prepared from the identified DESs ZnCl_2_‐AAc_2_, ZnCl_2_‐HEA_2_, ZnCl_2_‐DMAAm_2_ as monomers, BAPO or BDEABP as photoinitiator, and small amounts of covalent crosslinker PEGDA M_n_ 700 as indicated for each case. The presence of very low amounts of PEGDA ensures better stability of the printed structures during development while keeping the main features of the materials.

Once thoroughly characterized, the DESs were formulated into deep eutectic inks (DEIs). MPLP of the DEIs was performed using a commercial printing set‐up (Photonic GT2, Nanoscribe GmbH), which is based on a femtosecond laser at center wavelength *λ* = 780 nm. For this purpose, we first dissolved suitable photoinitiators known for efficient multiphoton polymerization such as phenylbis(2,4,6‐trimethylbenzoyl)phosphine oxide (BAPO) or bis(diethylamino)benzophenone (BDEABP) directly in the DESs (**Figure** **3** and Experimental Section). To investigate the printing performance of the optimized DEIs, we 3D printed benchmark boat microstructures (dimensions of 50 µm × 25 µm × 40 µm) with varying laser power and scanning speed (Figure S17, Supporting Information). Even without the addition of any covalent crosslinkers, micrometer‐scale buckyballs with dimensions of 54 µm × 54 µm × 50 µm were successfully printed using a laser power of 20 mW and scanning speed of 40 mm s^−1^ (see Video S1 and Figure S18, Supporting Information).

Nevertheless, we found that the presence of very low amounts of covalent multifunctional crosslinker, i.e., PEGDA‐M_n_ 700 (4‐5 wt%), in the DEIs was beneficial to ensure the stability of the printed structures throughout development and in different media. Thus, we maintained a constant molar ratio of 86.5:1 employed acrylic monomer in the deep eutectic mixture to covalent multifunctional crosslinker PEGDA‐M_n_ 700 in the final DEIs (Figure 3). The three prepared DEIs exhibited a broad printability window allowing exceptional printing speeds (>20 mm s^−1^) at low laser powers (10‐15 mW) (Figures S19–S21, Supporting Information), respectively. It should be noted that, in contrast to the frequently employed multifunctional monomers such as tri‐ or tetra‐acrylates, the DEIs contained only very low amounts of covalent multifunctional photopolymerizable crosslinker (4‐5 wt%). Nevertheless, the DEIs printability was found to reach the printing performance of these commonly used, highly sensitive materials for MPLP such as, for example, the commercially available IP‐S (Nanoscribe GmbH) or of a custom‐made ink composed of 99.5 wt% pentaerythritol triacrylate (PETA) and 0.5 wt% 7‐diethylamino‐3‐thenoylcoumarin (DETC) (Figures S22 and S23, Supporting Information). This suggests that zinc chloride forms photocrosslinkable entities with acrylic monomers in the DESs mimicking the behavior of multifunctional photopolymerizable crosslinkers during printing. For example, a comparative ink with similar molar ratios, but without zinc chloride (Tables S1 and S2, Supporting Information), was not printable using the same range of printing parameters as depicted in Figure S24 (Supporting Information) and showed only a very narrow printability window leading solely to unstable microstructures even when using high laser power and low scanning speed (Figure S25, Supporting Information). The printability as well as the resolution of the printed DEIs was found to be similar or better compared to that of the commercially available soft material ink IP‐PDMS (Nanoscribe GmbH) (see Table S3 and Figures S27–S30, Supporting Information).

To study the shape fidelity of the printed DEIs in water, we 3D printed free‐standing net microstructures with dimensions of 25 µm × 25 µm × 10 µm and analyzed their deviations from the model by using confocal fluorescence microscopy. Quantitative analysis of the printed net microstructures revealed excellent shape fidelity for DEI1 and DEI2. The printed DEI3 showed minor relative deviations of ≈10% by swelling in water indicating hydrophilic properties (see Table S4 and Figure S31, Supporting Information).

To demonstrate printability, the three DEIs formulations were also used for the fabrication of complex free‐standing larger millimeter‐sized 3D structures such as an “Eschenheimer Turm” printed with DEI1, “Eiffel tower” printed with DEI2, or “Michelangelo's David” printed with DEI3 (Figure 3). The 3D‐printed structures showed excellent shape fidelity as evidenced by the recorded SEM images. Furthermore, to show the stability of a mechanically highly demanding, delicate structure in water, we added rhodamine B methacrylate (0.01 wt% of total ink mass) as a fluorescent dye to DEI1 to perform confocal fluorescence microscopy after printing. This enabled, for example, the confocal imaging and 3D construction of a 3D microprinted “Atomium” with dimensions of 600 µm × 600 µm × 600 µm in aqueous media (Video S2, Supporting Information).

### 2.3 In‐Depth Characterization of Printed Material

The resulting chemical composition of the printed materials was investigated spectroscopically and thermogravimetrically (see Supporting Information). The development process with isopropanol and water was found to remove almost all zinc ions resulting in a very low crosslinked network by breaking the supramolecular ion‐dipole and hydrogen bonds. Residual traces of zinc ions could be successfully removed by employing an additional development step with a chelat binding ligand solution. Thus, the presence of zinc chloride plays a crucial role in the printability, but also can be easily removed having an impact on the final properties of the 3D printed materials.

**Figure 4:**
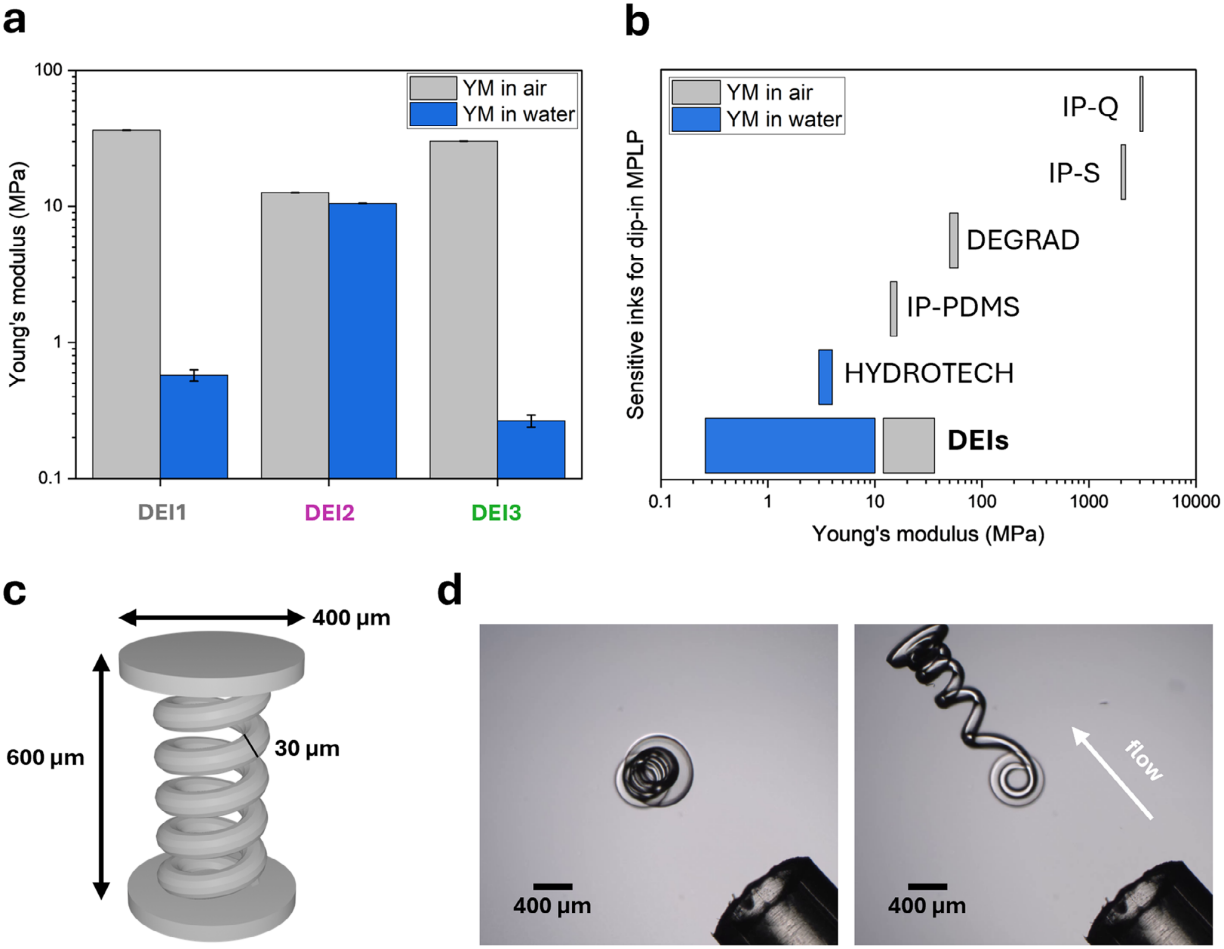
Mechanical characterization of the printed DEIs. The mechanical properties have been investigated quantitatively by compression experiments and qualitatively by flow‐induced stretching/bending. a) Young's moduli of 3D printed DEIs in the dry state and in water have been determined by studying the compression of printed pillars with 400 µm diameter and 300 µm height, respectively. The Young's modulus has been calculated from force‐strain displacement curves in the linear regime (see Tables S5 and S6, Supporting Information). b) Comparison of mechanical properties of different available material inks for dip‐in mode MPLP. DEIs extend the range of accessible materials to the range of low Young's moduli (Table S9, Supporting Information).^[^ ^50^, ^51^, ^52^ ^]^ c) 3D model of spring with dimensions of 600 µm in height, 100 µm in diameter, and 30 µm in thickness. d) 3D printed helical spring has been subjected to a water flow induced by a microliter pipette (Video S4, Supporting Information). The optical microscopy images have been recorded without flow (left) and with flow (right).

As a next step, we studied the mechanical properties of the 3D printed DEIs using a custom‐built compression set‐up for measurements in the dry state and in water. For this purpose, we printed and analyzed cylindrical pillars of 400 µm in diameter and 300 µm in height (**Figure** **4** and Supporting Information).

The printed DEIs demonstrated a broad, tuneable range of Young's moduli in compression, ranging from 36 MPa in the dry state to 0.26 MPa in aqueous media (see Figure 4; Figures S37–S48 and Tables S5–S8, Supporting Information). These values are attributed to the low covalent crosslinker concentrations in the DEI formulations, which enable much softer mechanical properties than those observed in current commercial dip‐in‐compatible inks (Figure 4; Table S9, Supporting Information). For example, commercial inks such as IP‐Q and IP‐S exhibit Young's moduli in the GPa regime (3100 and 2100 MPa, in the dry state respectively). Compared to softer materials such as DEGRAD (50–60 MPa) and particularly to IP‐PDMS (15.3 MPa), DEIs offer a more versatile material platform, covering a wider mechanical spectrum in the low‐modulus regime, while avoiding the limitations of hydrophobic, PDMS‐based chemistries. DEIs exhibited Young´s moduli between 0.26 and 10 MPa under aqueous conditions. The significant variation in Young's modulus—spanning over two orders of magnitude—between the dry state and in water can be explained by material swelling. For the hydrophilic DEI1 and DEI3, based on poly(acrylic acid) and poly(dimethylacrylamide), Young's moduli in water were notably lower. In contrast, DEI2 showed similar Young's moduli both in air and in water. The printed pillars were compressible up to strain of 40% without observing any defects (Video S3, Supporting Information). It should be noted that conventional hydrogel inks often suffer from bad mechanical stability and are not comparable with DEI systems in terms of printability performance. To the best of our knowledge, the only commercially available hydrogel ink compatible with the dip‐in mode for printing larger structures is HYDROTECH INX© X200, which exhibits a Young Modulus ≈3–4 MPa in aqueous media. However, DEIs not only cover this range but also reach significantly lower stiffness values in water—down to 260 kPa—thereby enabling the fabrication of highly compliant, responsive microstructures as demonstrated below.

To demonstrate the elastic material properties, we printed free‐standing helical spring structures of DEI1 with dimensions of 600 µm in height, 100 µm in diameter, and 30 µm in thickness. After placing the springs in water, we induced a periodic flow with a microliter pipette (Figure 4; Video S4, Supporting Information). We observed a significant bending response of the spring. Stopping the flow resulted in the recovery of the 3D‐printed initial state confirming the elasticity of the 3D‐printed DEI.

### 2.4 Extending Functionality

**Figure 5:**
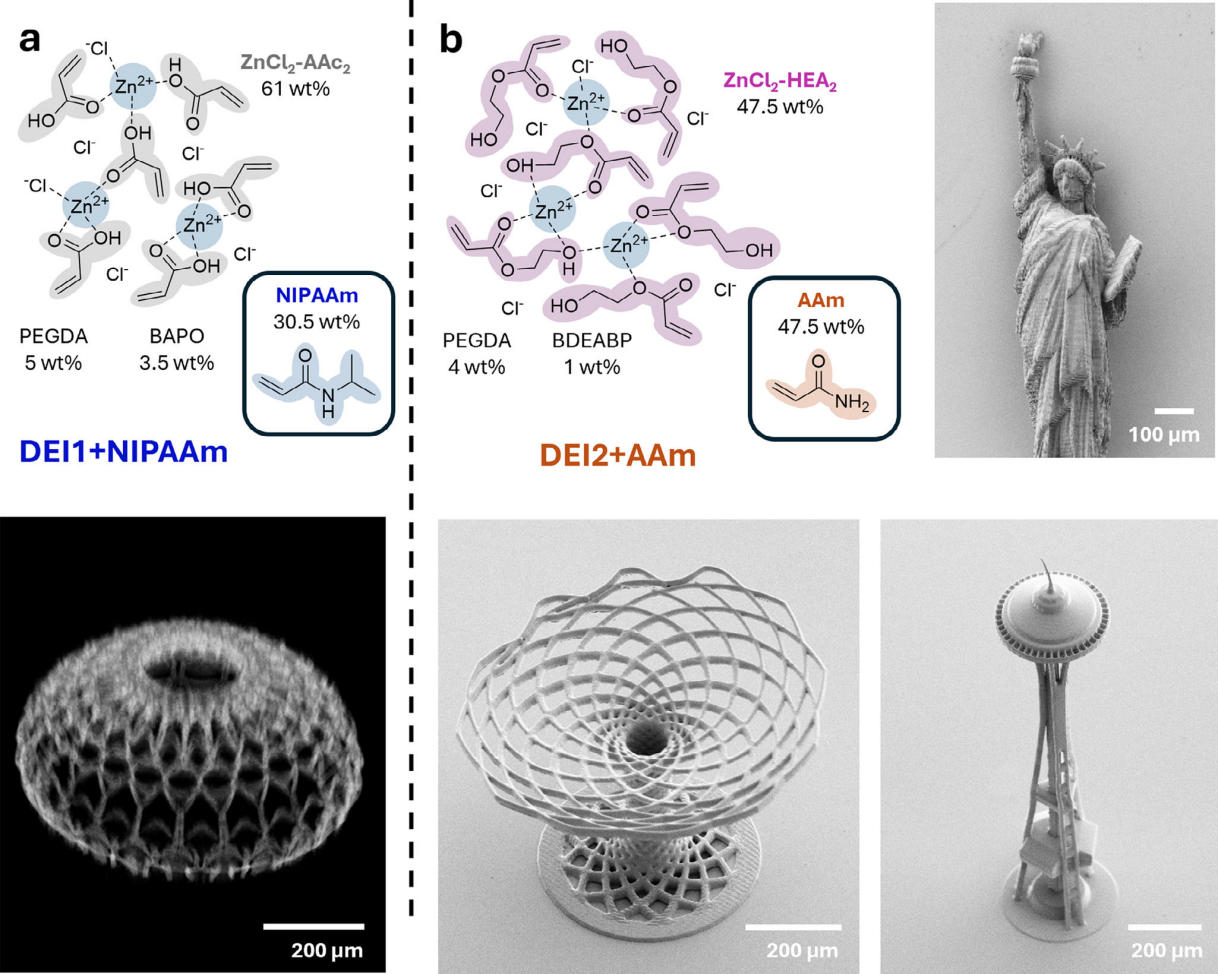
Functional 3D microstructures from DEIs. Further functionalities are introduced by mixing the different DEIs with other acrylic monomers. To prove the versatility, we have combined two identified DEIs, i.e., DEI1 and DEI2, with *N*‐isopropylacrylamide (NIPAAm) – a well‐known monomer leading to thermoresponsive polymers – or with acrylamide (AAm), respectively. a) DEI1+NIPAAm (top) has been employed to print, for example, the “Al Wasl Dome”. A 3D reconstruction of the dome structure has been obtained using confocal fluorescence microscopy and verified its mechanical stability (bottom). Furthermore, the temperature‐response of DEI1+NIPAAm has been studied in printed buckyball microstructures as well as multimaterial microgrippers (see Supporting Information). b) DEI2+AAm (top) has been successfully used for 3D microprinting of mechanically demanding structures such as, for example, the “Statue of liberty”, “Seattle space needle”, or the “Albero della vita” with high shape fidelity. SEM images of these printed structures are shown (right and bottom).

We further demonstrated the versatility of the new ink design approach by first mixing the DESs with other monofunctional monomers. This allowed, for example, for the preparation of multicomponent DESs with monomers that could not be used with zinc chloride alone. For example, we combined the previously identified DESs with *N*‐isopropyl acrylamide (NIPAAm) as well as acrylamide (AAm) (see Tables S10 and S11, Supporting Information). Both monomers showed good solubility in the identified DESs and were used for the preparation of the two DEIs, i.e., DEI1+NIPAAm and DEI2+AAm (**Figure** **5**). Both exhibited printability in a printing parameter range similar to the three previously identified DEIs (Figures S50 and S55, Supporting Information). Printed DEI1+NIPAAm showed the expected multiresponsive behavior and could also be combined with other DEIs in multimaterial structures offering, for example, targeted directional actuation of responsive 3D structures (see Videos S5 and Video S6, and Figures S51–S54, Supporting Information). Furthermore, the addition of rhodamine B methacrylate (0.01 wt% of total ink mass) to DEI1+NIPAAm allowed for confocal fluorescence imaging of a delicate 3D printed “Al Wasl Dome” structure with dimensions of 600 µm × 600 µm × 400 µm (Video S7, Supporting Informations; Figure 5). DEI2+AAm was used to print a millimeter‐scaled “Statue of liberty,” the “Seattle space needle,” and the “Albero della vita” with a laser power of 50 mW and scanning speed of 20 mm s^−1^ (Figure 5; Figure S56, Supporting Information). The recorded SEM images confirm the high quality and stability during printing of the mechanically demanding, very thin, and delicate structures. The optical microscopy images (Figure S57, Supporting Information) of the printed statue showed several fine detailed features such as the crown, face, and fire of the statue. Inspired by buildings constructed for the world exhibition, we also printed the structurally demanding “Atomium”. SEM images of this microarchitecture are included (Figure S58, Supporting Information).

## 3 Conclusion

The use of DESs in MPLP is proved to be an excellent approach to overcome existing limitations in combining the desired excellent printability of highly crosslinked materials with targeted soft mechanical properties. This goal is achieved by exploiting the present supramolecular interactions in the printing process which are later easily broken in the subsequent development step. Following this approach, the identified and presented DEI models exhibit exceptional printability performances with only a very low and tunable amount of covalent multifunctional crosslinker. This leads to a very low crosslinking density, which results in soft and elastic mechanical properties as well as significantly increasing responsive behavior in 4D microprinting applications. Furthermore, by adding other functional monomers, the functionality of the printed (multimaterial) structures can be easily extended while maintaining excellent printability.

Future efforts will effectively integrate DESs materials into applications in soft robotics, cell scaffold fabrication, metamaterial design, optics and photonics, sensors, and micro‐electromechanical systems. In addition to that, we believe our work on DEIs for 3D microprinting represents just the beginning of a wealth of new possibilities for ink design. The versatility and simplicity of the ink preparation significantly facilitates its handling for potential users across disciplines, thus ensuring its broad applicability.

## 4 Experimental Section

### Materials

All chemicals and solvents were purchased from commercial suppliers and used without prior purification. In detail, zinc (II) chloride was purchased from TCI chemicals. Acrylic acid (AAc), 2‐hydroxyethyl acrylate (HEA), polyethylene glycol diacrylate (M_n_ 700), *N,N*‐dimethylacrylamide, BAPO, BDEABP, zincon, EDTA, and polyacrylic acid (M_n_ 20 000) were purchased from Merck. *N‐*isopropylacrylamide (NIPAAm) and acrylamide (AAm) were purchased from BLDpharm. HPLC‐grade isopropanol and water used in this work were purchased from Fisher Scientific. All chemicals, mixtures, inks, and samples were stored and handled under yellow light.

### Preparation of DESs

DESs were prepared by adding the acrylic monomer to a flask with zinc (II) chloride in varying molar ratios. The prepared batches typically ranged from 5 g to 12 g by mass in total. The flask was heated to 90 °C for 30–60 min and vigorously stirred until a homogeneous, transparent, and liquid mixture was afforded. The obtained DESs remained liquid after cooling to room temperature and were used for the preparation of the ink.

### Characterization of DESs

DSC was performed to characterize the deep eutectic behavior using the DSC 250 of TA Instruments in a temperature interval between 40 and −80 °C. The shown data is recorded from the second heating cycle.

The flow properties of the DESs were measured by rotational rheometry using a HAAKE MARS rheometer. The shear rate was varied between 1 and 1000 s^−1^ in 30 steps. The measured viscosity at 1000 s^−1^ is described in the results section.

1H NMR spectra were recorded using a Bruker Avance III 300 NMR spectrometer at room temperature and 300 MHz. The acrylic monomers were recorded in CDCl_3_ as a solvent. The DESs were recorded in WILMAD NMR tubes with a coaxial insert tube filled with CDCl_3_.

Refraction indices were determined at 20 °C for varying wavelengths using a Schmidt+Haensch ATR L refractometer. UV‐vis spectroscopy and FT‐IR of the deep eutectic mixtures were measured with a Jasco V‐770 and a Jasco FT/IR‐4600 spectrometer, respectively.

### Preparation of DEIs

DEIs were prepared by mixing the DESs of interest with PEGDA as a covalent crosslinker in a molar ratio of 0.0116 mol% (4‐5 wt%) polymerizable molecules. The photoinitiator, i.e., BAPO or BDEABP, was selected based on solubility in the DEI and added in a concentration of 3.5 or 1.0 wt%, respectively. Rhodamine B methacrylate was added to the DEI in a concentration of 0.01 wt% to achieve covalent incorporation of a fluorescent dye for confocal microscopy imaging.

### Silanization Procedure

Glass coverslips (Marienfeld, 170 ± 5 µm) were washed with isopropanol and acetone and dried with pressurized nitrogen. Subsequently, the surface of all slides was activated for 1 min by plasma treatment using a TDK PiezoBrush. Afterward, the coverslips were immersed in a 4 mM solution of 3‐(trimethoxysilyl)propyl acrylate in toluene for 1.5 h. Last, the acrylate‐functionalized coverslips were washed twice in toluene and once in acetone and used as substrates for microprinting.

### Multiphoton 3D Laser Printing

MPLP was performed with a commercially available set‐up (Photonic Professional GT2, Nanoscribe GmbH & Co. KG) using a 63× objective (NA = 1.4), 25× objective (NA = 0.8) or 10× objective (NA = 0.3) from Zeiss for focusing the femtosecond laser with a center wavelength of 780 nm. All microstructures were printed using an oil immersion (63× and 25×) or dip‐in (10×) configuration. The procedure reported by Toulouse et al. was used for the 10× objective.^[^ ^53^ ^]^ Before printing, GWL files were generated with the Describe software (Nanoscribe GmbH) from previously designed STL files of desired geometries by setting slicing and hatching to 300 nm (25×) or 5 µm and 1 µm (10×) for all printed geometries, respectively. The structures were developed in isopropanol or propylene glycol methyl ether acetate depending on the printed material to remove uncured ink. After development, the printed structures were subjected to air drying before analysis. The printing parameters for the shown demonstrating structures are included in the Supporting Information.

### Optical Microscopy

Optical images were recorded with a Zeiss Axio Imager M2 microscope using a 5× long‐distance Zeiss objective (NA = 0.13) or a LD Plan‐Neofluar 20×/0.4 Korr Ph M27 objective (NA = 0.4) and an Axiocam 705 microscope camera.

### Scanning Electron Microscopy

All SEM images were acquired using a Zeiss Supra 55VP (Carl Zeiss AG) instrument at 3–5 kV in secondary electron mode. Prior to imaging, the structures were sputter coated with a Pt–Pd layer of 12 nm. The structures were imaged from in top view or in side view (40°).

### Confocal Laser Scanning Microscopy

Confocal fluorescence microscopy was performed using a Nikon A1R confocal microscope (10×, NA  =  0.45 and 20×, NA  =  0.75) equipped with GaAsP‐detectors by exciting the covalently incorporated rhodamine B methacrylate dye with 561 nm laser and detecting the fluorescence at 595 nm. To enable confocal fluorescence imaging of DEI1 or DEI3, rhodamine B methacrylate was added as 0.01 wt% to the total ink mass. DEI2 was imaged using an excitation laser wavelength of 488 nm. Confocal imaging of printed IP‐PDMS was performed using an excitation laser wavelength of 404 nm. The Atomium structure was recorded in HPLC grade water and the “Al Wasl Dome” structure was recorded in a solution of 70% ethanol and 30% HPLC water.

### Analysis of Temperature‐Responsive Behavior

Heating of printed structures was performed with a heating stage (LTS 420, Linkam Scientific Instruments) in the optical microscope. The transmission illumination mode was used for imaging. The investigated temperature program started at 25 °C and heated the *N*‐isopropylacrylamide containing printed structures up to 60 °C using a gradient of 5 °C min^−1^. The heated microstructures were subsequently cooled using a gradient of 5 °C min^−1^ back to room temperature. The swelling factor S_V_ was calculated by estimating the volume from the top area of the printed structure.

### Analysis of pH‐ and Calcium‐Responsive Behavior

Calcium‐responsive behavior was analyzed by washing the 3D printed structures several times with a calcium chloride solution (0.24 g mL^−1^). The pH response was analyzed by washing the structures several times with an aqueous sodium hydroxide solution with a pH of 10.6.

### Characterization of Chemical Composition of 3D Printed DEI1

FT‐IR spectra of 3D printed cubes (50 µm × 50 µm × 20 µm) were acquired using a Bruker Lumos II and averaged for five structures. UV‐vis spectra of the Zincon solution (1 mL, 188 µM) were recorded with a Jasco V‐770 spectrometer. Thermogravimetry (TGA) of 3D printed cubes (500 µm × 500 µm × 1 mm) was performed in a nitrogen flow (50 mL min^−1^) using a TGA 2 of Mettler Toledo and heating from 30 to 600 °C with rate of 10 °C min^−1^.

### Mechanical Characterization of Printed DEIs

The mechanical characterization of the presented materials was performed by conducting uniaxial‐loading experiments using a home‐built setup (Figure S35A, Supporting Information). This setup was previously used in slightly different configurations.^[^ ^54^, ^55^ ^]^

For each material, six pillar samples were printed on glass coverslips (Marienfeld, 170 µm ± 5 µm) with dimensions of 300 µm in height and 400 µm in diameter with the following printing parameter using the 10× objective and dip‐in configuration: laser power 100%, scanning speed 20 mm s^−1^, slicing 5 µm, and hatching 1 µm.

To prevent warping of the substrates during the measurements, all substrates were mounted onto cleaned soda‐lime glass (Nanoscribe GmbH & Co. KG, 25 mm × 25 mm × 0.7 mm) and fixed via a thin layer of glue (UHU, Plast Special), distributed with a syringe. After mounting, the samples were air‐dried for 24 h. Samples measured in aqueous media required additional preparation steps; therefore, optical transparent cuvettes (Brand GmbH & Co. KG, 12.5 mm × 12.5 mm × 45 mm) were cut into pieces (12.5 mm × 12.5 mm × 3 mm) and coated on the underside with UV‐glue (Best Uvirapid 702, BEST Klebstoffe). The UV‐glue allows for alignment and centering the cuvette pieces around the pillar arrays on the substrates, before curing and sealing them with an UV‐LED flashlight for 2 min (Figure S35C, Supporting Information).

To obtain the effective spring constant and the Young's modulus of the materials, four out of six pillars for each material were measured as follows:

The substrate was placed and tightly fixed on the sample holder underneath the stamp. The stamp itself was centered around a pillar and loaded the pillar by a prescribed displacement normal to the substrate surface, gradually moving down along the *z*‐direction by the translation stages. After reaching the maximum displacement, the stamp unloads the sample. During loading and unloading, the force is recorded by the force sensor. Loading and unloading cycles are repeated ten times with a delay time of 40 s between each cycle. To ensure sufficient initial contact between the stamp and the sample, the alignment is observed by the cameras. An illustration of a measurement cycle can be seen in Video S3 (Supporting Information).

For the measurements in aqueous media, the samples were stored 2 h in deionized water before conducting the experiments. In between the measurements, evaporated water was replaced with a pipette to prevent drying out of the samples.

## Conflict of Interest

Philipp Mainik, Christoph A. Spiegel, and Eva Blasco have filed a patent application on deep eutectic inks for MPLP.

## Supporting information



Supporting Information

Supplemental Video 1

Supplemental Video 2

Supplemental Video 3

Supplemental Video 4

Supplemental Video 5

Supplemental Video 6

Supplemental Video 7

## Data Availability

The data that support the findings of this study are openly available in heiData, a data repository of Heidelberg University at https://doi.org/10.11588/DATA/UP26J7, reference number 1011588.
