# Supplementary material for: Deep Eutectic Inks for Multiphoton 3D Laser Microprinting
Source: Adv Mater. 2025 Jun 25;37(38):2507640. doi: 10.1002/adma.202507640 (PMC12464625; doi:10.1002/adma.202507640)
Supplement: Supplementary file 1 — Supporting Information [file ADMA-37-2507640-s007.docx]

Supporting Information

Deep Eutectic Inks for Multiphoton 3D Laser Microprinting

Philipp Mainik, Christoph A. Spiegel, Jonathan L.G. Schneider, Martin Wegener, Eva Blasco*

P. Mainik, C. A. Spiegel, E. Blasco

Institute for Molecular Systems Engineering and Advanced Materials (IMSEAM), Heidelberg University, 69120 Heidelberg, Germany
E-mail: eva.blasco@uni-heidelberg.de

P. Mainik, C. A. Spiegel, E. Blasco

Institute of Organic Chemistry (OCI), Heidelberg University, 69120 Heidelberg, Germany

J. L.G. Schneider, M. Wegener
Institute of Applied Physics, Karlsruhe Institute of Technology (KIT), 76131 Karlsruhe, Germany

M. Wegener

Institute of Nanotechnology, Karlsruhe Institute of Technology (KIT), 76344 Eggenstein-Leopoldshafen, Germany.

Table of Contents

| Differential scanning calorimetry of identified DESs  Rotational rheology of identified DESs  NMR spectroscopy of identified DESs  Optical properties of identified DESs  Printing of DEIs without additional crosslinker  Printability window of optimized DEIs  Printability windows of highly sensitive reference systems (IP-S, PETA)  Printability of optimized DEI and zinc-free comparative ink  Printability window of reference soft material ink (IP-PDMS)  Resolution tests  Shape fidelity of printed structures in water  Optical microscopy image of printed DEI1  Printing parameters for the shown buildings and statues  FTIR spectroscopy of 3D printed DEI1  Analysis of residual zinc ions  Mechanical characterization of printed DEIs  Comparison of mechanical properties with available inks for dip-in mode MPLP  Extending functionality by mixing DEIs with N-isopropyl acrylamide  3D printed temperature-, pH-, and calcium-responsive DEI1+NIPAAm  Extending functionality by mixing DEIs with acrylamide  SEM images of 3D printed cubic grids  References | 3  5  7  11  14  15  17  18  20  21  24  26  27  28  30  32  43  44  45  48  51  52 |
| --- | --- |

Differential scanning calorimetry of identified DESs

DSC measurements of ZnCl_2_-AAc*_x_* mixtures with varying molar ratio *x* were performed and compared to thermograms of the starting compounds, *i.e.* acrylic acid and zinc chloride. The characteristic peak in the thermogram at the melting temperature of acrylic acid (*T*_m_ = 10 °C) vanishes in the deep eutectic mixtures. Instead, a new glass transition appears in a temperature range between -66.4 °C to -45 °C.


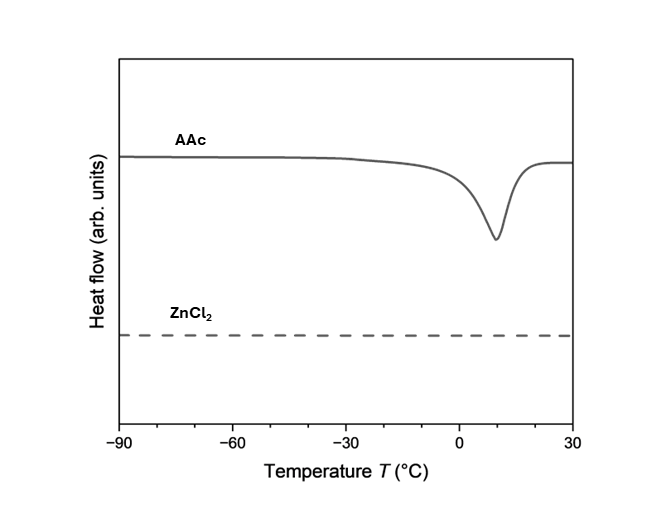


**Figure S1:** DSC thermogram of the starting compounds ZnCl_2_ and acrylic acid. The stack is shown with an offset. The melting point of AAc vanishes in the DSC thermograms of the ZnCl_2_-AAc*_x_*.


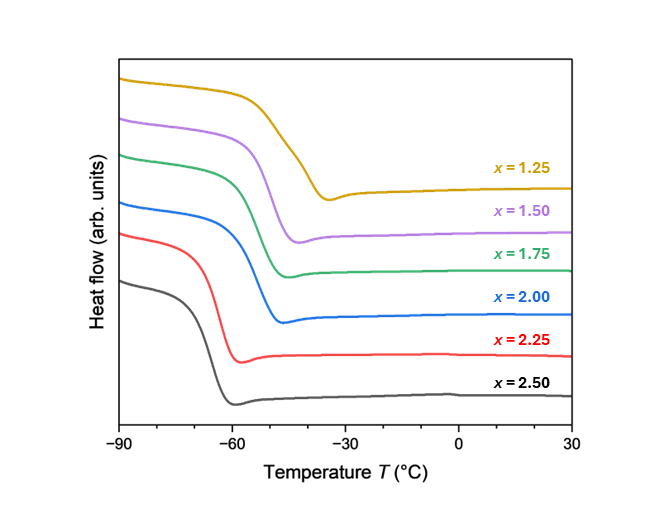


**Figure S2:** DSC thermogram of ZnCl_2_-AAc*_x_* with varying molar ratio *x* from 1.25 to 2.50. The stack is shown with an offset.

Rotational rheology of identified DESs

Rheological analysis of ZnCl_2_ and acrylic acid containing DESs was performed by rotational rheology. Increasing the molar ratio *x* of the acrylic acid in the ZnCl_2_-AAc*_x_* systems allowed for tuning the dynamic viscosity in a range between 2×10^4^ mPa s to 2×10^2^ mPa s.


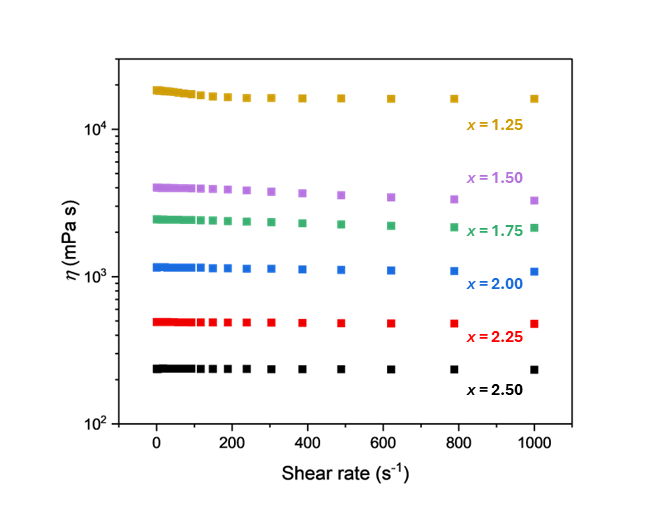


**Figure S3:** Rotational rheology measurements. Dynamic viscosity of DESs ZnCl_2_-AAc*_x_* for varying shear rates. Higher molar ratios *x* reduced the viscosity of the DES.


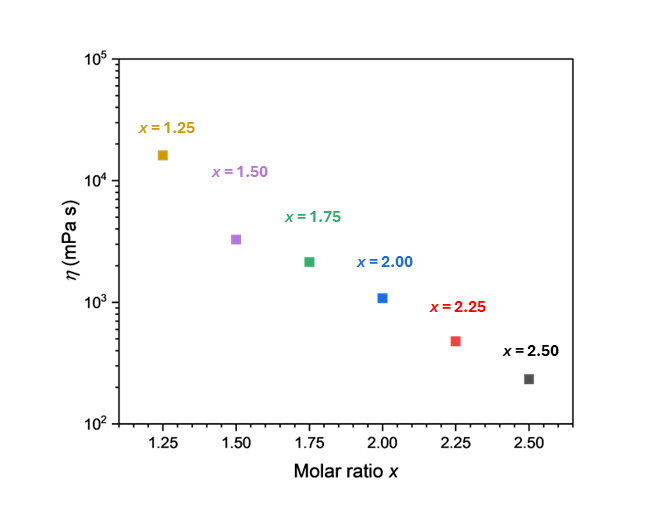


**Figure S4:** Tunable dynamic viscosity depending on the molar ratio *x* in ZnCl_2_-AAc*_x_*. The viscosity was determined at a shear rate of 1000 Hz by rotational rheology, respectively.


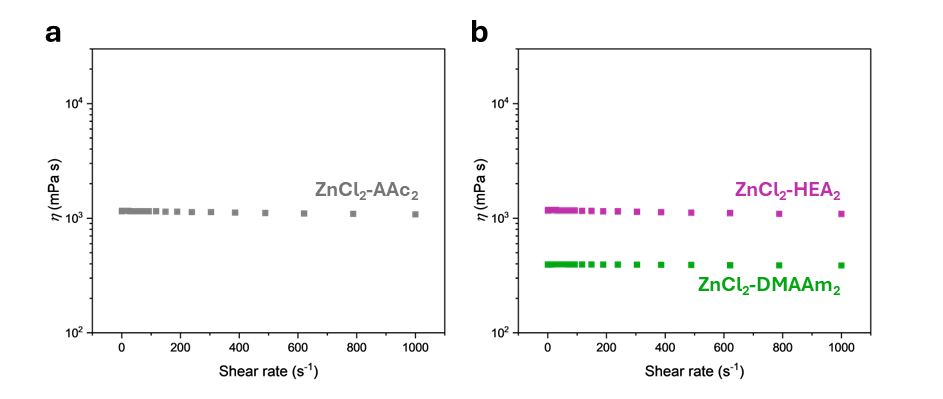


**Figure S5:** Dynamic viscosities of the three DESs for varying shear rates from rotational rheology measurements. ZnCl_2_-AAc_2_ (in **a**) and ZnCl_2_-HEA_2_ (in **b**) show similar viscosities. ZnCl_2_-DMAAm_2_ shows lower viscosities compared to the other two systems.

NMR spectroscopy of identified DESs

^1^H NMR spectroscopy was performed of the starting acrylic monomers and the identified DESs. The acrylic monomers were dissolved and measured in CDCl_3_. The DESs were measured using a coaxial insert tube filled with CDCl_3_. In general, the DESs showed significant broadening of the ^1^H NMR bands which can be related to the increase in viscosity.


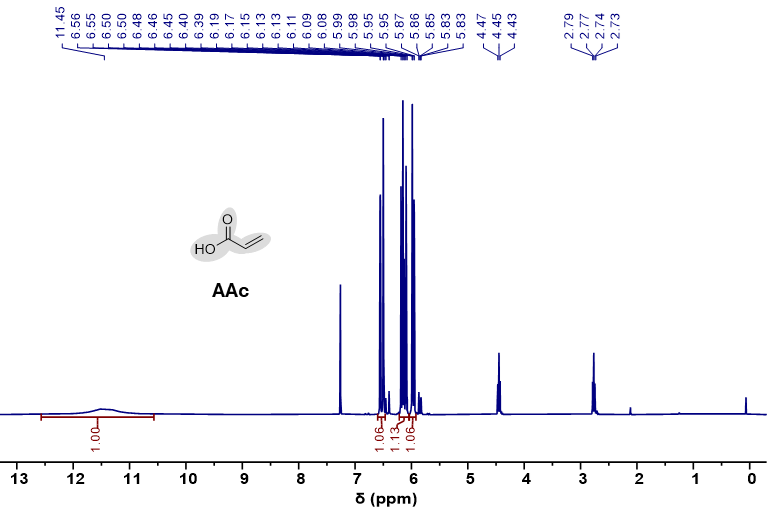


**Figure S6:** ^1^H NMR spectrum (300 MHz, CDCl_3_, room temperature) of acrylic acid.


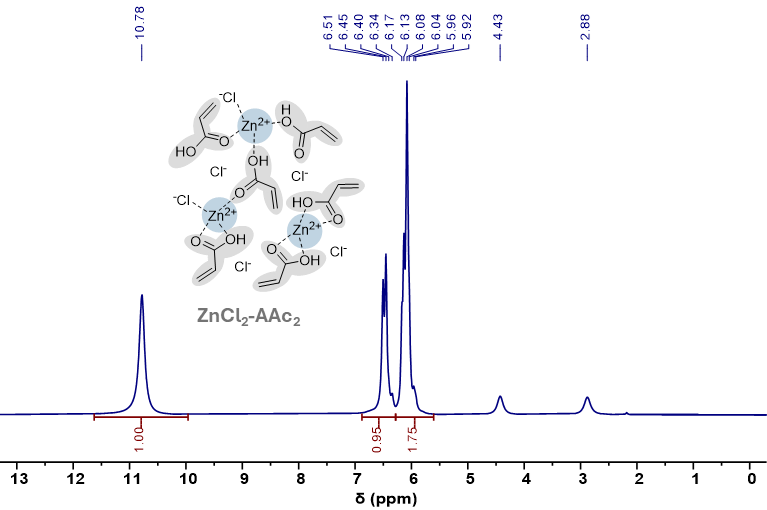


**Figure S7:** ^1^H NMR spectrum (300 MHz, CDCl_3_, room temperature) of ZnCl_2_-AAc_2_.

The δ-shift of the hydroxy proton in ZnCl_2_-HEA_2_ (compared to HEA) indicates a coordination of hydroxy groups to zinc ions (see **Figure S8** and **Figure S9**).


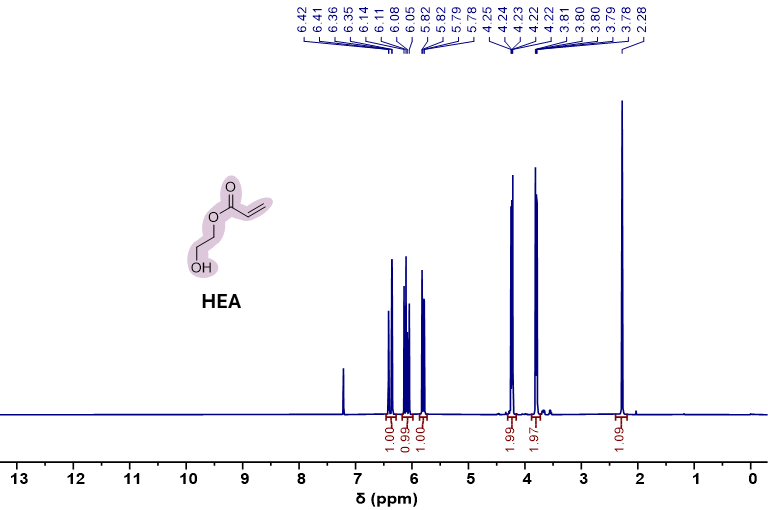


**Figure S8:** ^1^H NMR spectrum (300 MHz, CDCl_3_, room temperature) of 2-hydroxyethyl acrylate.


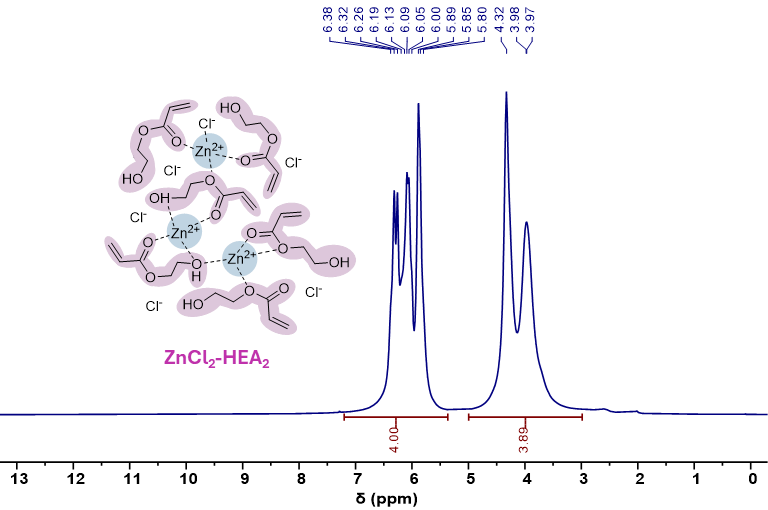


**Figure S9:** ^1^H NMR spectrum (300 MHz, CDCl_3_, room temperature) of ZnCl_2_-HEA_2_. In the DES, the signal for the hydroxy group is shifted towards higher ppm.

The doublet splitting of the methyl groups in ZnCl_2_-DMAAm_2_ (compared to DMAAm) indicate a strong coordination and polarization of the amide bond (see **Figure S10** and **Figure S11**).


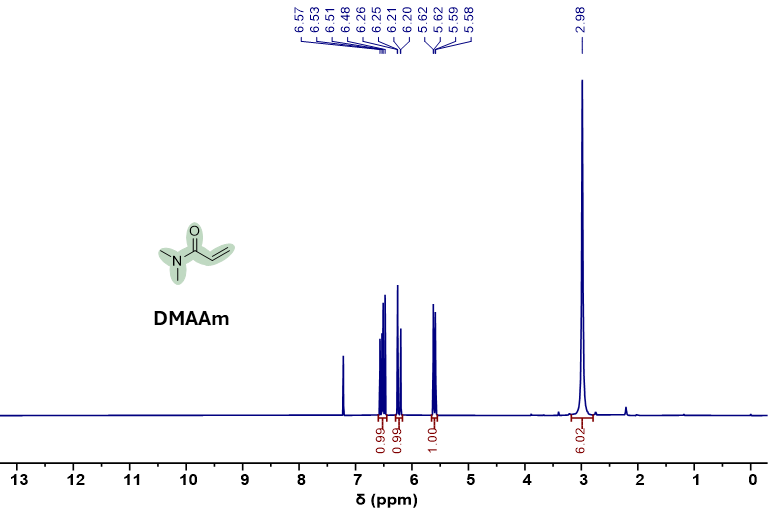


**Figure S10:** ^1^H NMR spectrum (300 MHz, CDCl_3_, room temperature) of *N*,*N*-dimethylacrylamide.


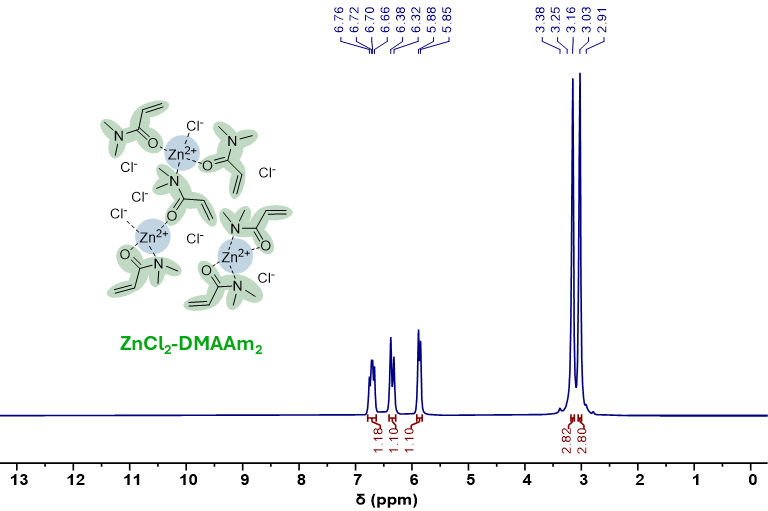


**Figure S11:** ^1^H NMR spectrum (300 MHz, CDCl_3_, room temperature) of ZnCl_2_-DMAAm_2_. The two methyl group signals are split in the DESs due to the strong polarization of the amide bond.

Optical properties of identified DESs

The three identified DESs ZnCl_2_-AAc_2_, ZnCl_2_-HEA_2_, and ZnCl_2_-DMAAm_2_ were analyzed for their optical properties such as refractive indices and absorption in the UV-vis and IR spectral range.

First, the refractive indices were measured using an ATR refractometer. The measured refractive indices of the three studied DESs (*η*_20°C_ = 1.48-1.52 at *λ* = 780 nm) are comparable to indices of various commercially available inks (IP-S, IP-Dip, IP-L, OrmoComp, IP-Visio, and PO4) frequently used in MPLP. ^[1]^ In detail, for ZnCl_2_-AAc_2_ and ZnCl_2_-HEA_2_, the refractive index at the center wavelength of the femtosecond pulsed laser *λ* = 780 nm was found to be similar with *η*_20°C_ = 1.479 and *η*_20°C_ = 1.489, respectively (**Figure S12**). The refractive index of ZnCl_2_-DMAAm_2_ at 780 nm was slightly higher with *η*_20°C_ = 1.519.


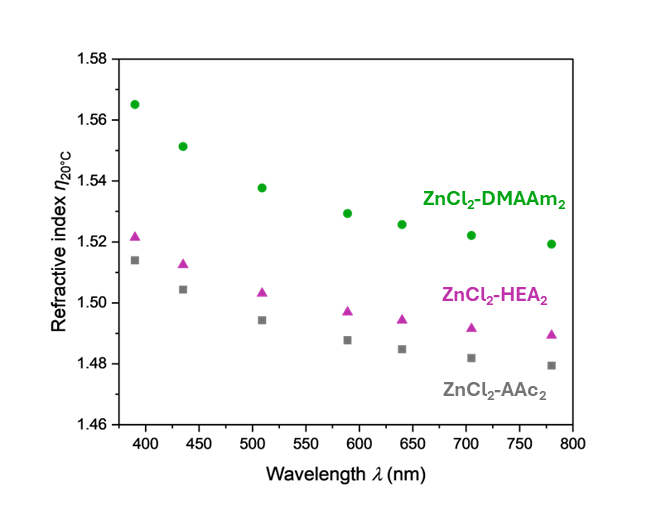


**Figure S12:** Refractive indices of investigated DESs at different wavelengths. The refractive indices are in a similar range compared to different commercially available inks such as IP-S, IP-Dip, IP-L, OrmoComp, IP-Visio, and PO4 which are frequently used in MPLP. ^[1]^

After characterizing the refractive optical properties, we analyzed the optical absorption of the identified DESs by UV-vis spectroscopy. The colorless mixtures ZnCl_2_-AAc_2_ and ZnCl_2_-HEA_2_ did not exhibit any absorption in the range between 350 nm and 900 nm. The mixture ZnCl_2_-DMAAm_2_ did not show absorption between 400 nm and 900 nm. The high optical transparency in the visible and near IR range around make these mixtures highly suitable for multiphoton 3D laser printing.


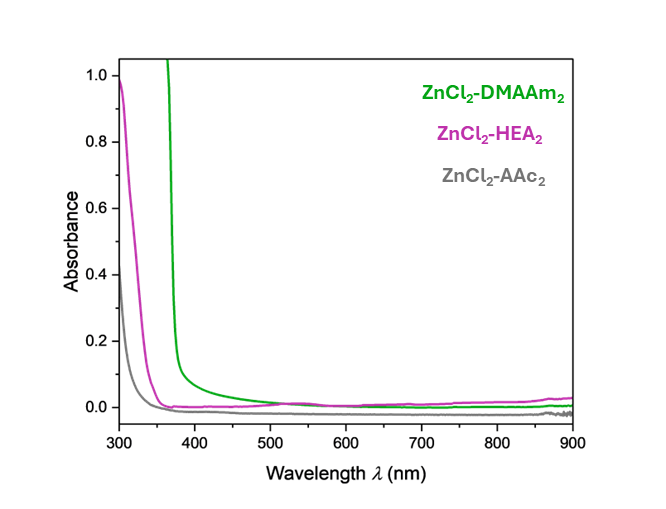


**Figure S13:** UV-vis spectra of investigated DESs. All DES are optically transparent and do not absorb light between 400 nm and 800 nm making them highly suitable for MPLP.

To extend the UV-vis analysis of the absorption properties to the IR spectral range, we have performed detailed FTIR spectroscopic analysis of ZnCl_2_-AAc_2_, ZnCl_2_-HEA_2_, and ZnCl_2_-DMAAm_2_ and compared them to the recorded spectra of pure monomers, *i.e.*, acrylic acid, 2-hydroxyethyl acrylate, and *N*,*N-*dimethylacrylamide, respectively (see **Figure S14** to **Figure S16).**


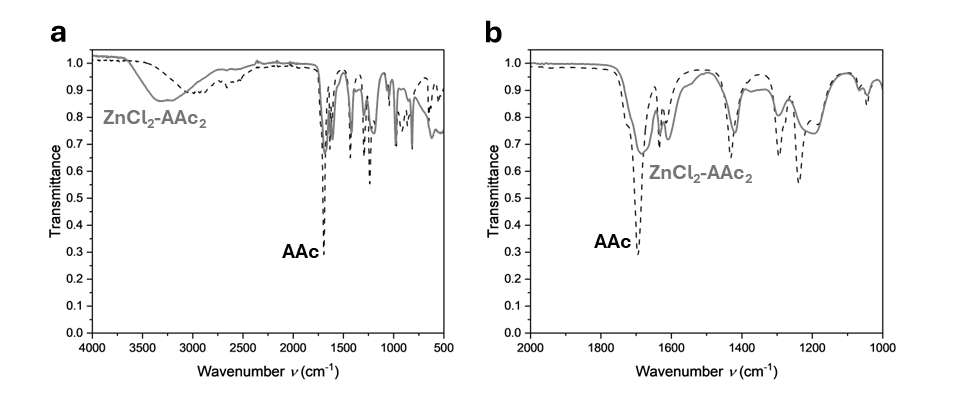


**Figure S14:** IR spectra of acrylic acid and ZnCl_2_-AAc_2_ in **a)** the full range from 4000 cm^-1^ to 500 cm^-1^ and **b)** a zoom-in from 2000 cm^-1^ to 1000 cm^-1^. The broadening of the peaks compared to the monomer can be explained by the dynamic species in DES.


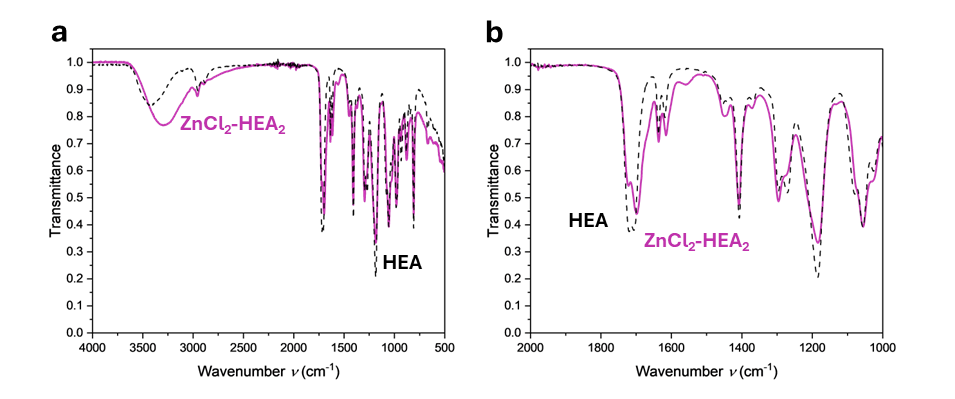


**Figure S15:** IR spectra of 2-hydroxyethyl acrylate and ZnCl_2_-HEA_2_ in **a)** the full range from 4000 cm^-1^ to 500 cm^-1^ and **b)** a zoom-in from 2000 cm^-1^ to 1000 cm^-1^. The broadening of the peaks compared to the monomer can be explained by the dynamic species in DES.


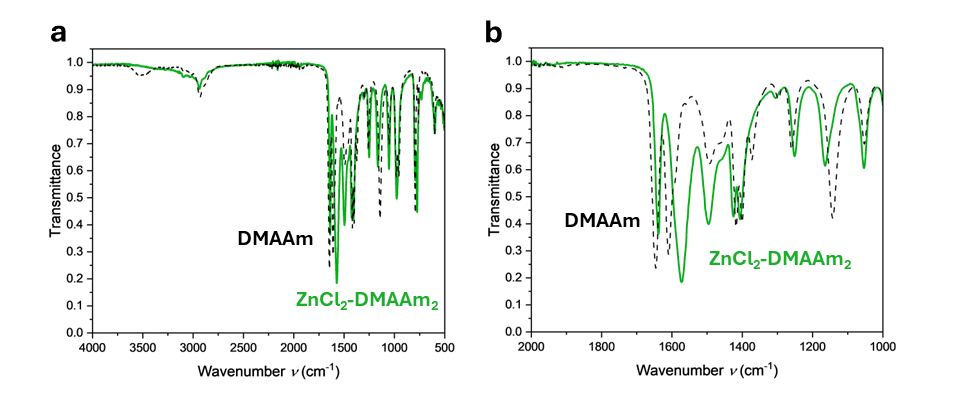


**Figure S16:** IR spectra of *N,N*-dimethylacrylamide and ZnCl_2_-DMAAm_2_ in **a)** the full range from 4000 cm^-1^ to 500 cm^-1^ and **b)** a zoom-in from 2000 cm^-1^ to 1000 cm^-1^. The broadening of the peaks compared to the monomer can be explained by the dynamic species in DES.

In the FTIR spectra, we identified three general trends. First, the DESs show an altered intensity ratio between the C=O carboxyl group and the C=C double bond in the acrylic moiety compared to the pure monomers. Second, a bathochromic shift of the C=O band indicates that the zinc ions act as a Lewis acid and interact with the oxygen of the carboxyl group, thereby weakening the C=O double bond. Furthermore, the observed broadening of the peaks in the FTIR spectra suggests that different species are formed by ion-dipole interactions in the three characterized DESs.

Printing of DEIs without additional crosslinker

To demonstrate that certain conditions allowed also the preparation of covalent crosslinker-free microstructures, we printed an ink (HEA-pure) composed of 99 wt% ZnCl_2_-HEA_2_ and 1 wt% DEABP. We have further examined various printing parameters such as laser power and scanning speed to evaluate the printability window for this covalent crosslinker-free ink (**Figure S17**). The printed microstructures were categorized in three different categories for their quality. Green printing parameter set allowed for high structural fidelity. Using the yellow printing parameters allowed for printing of the solid boat part but were limited for the fine boat features. Red printing parameters did not show stable structures or led to microexplosions due to local heat accumulation during printing.


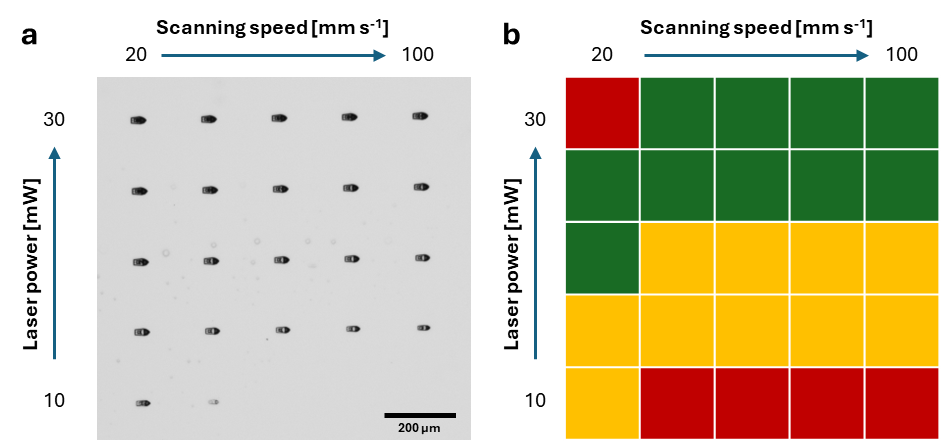


**Figure S17:** Printability window of a PEGDA-free ink composed solely of 99 wt% ZnCl_2_-HEA_2_ and 1 wt% DEABP for varying printing parameters. **a)** 3D printed benchmark boat structures (50 µm × 25 µm × 40 µm) and **b)** evaluation of printing parameters.

Moreover, we printed micrometric buckyballs using this covalent crosslinker-free ink (**Video S1**) and developed the microstructures in isopropanol. The 3D micrometric buckyballs did not dissolve and were imaged using optical microscopy (**Figure S18**).


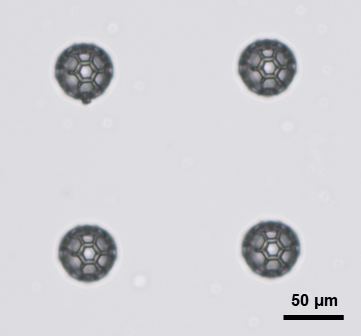


**Figure S18:** 3D printed buckyball (54 µm × 54 µm × 50 µm) with 99 wt% ZnCl_2_-HEA_2_ and 1 wt% DEABP after development in isopropanol (laser power 20 mW, scanning speed 40 mm s^-1^).

Printability window of optimized DEIs

The prepared and optimized DEIs were analyzed for their performance in MPLP. For this purpose, 3D benchmark boat microstructures with dimensions of 50 µm × 25 µm × 40 µm were printed with varying printing parameters, *i.e.* laser power and scanning speed using the 25× objective.


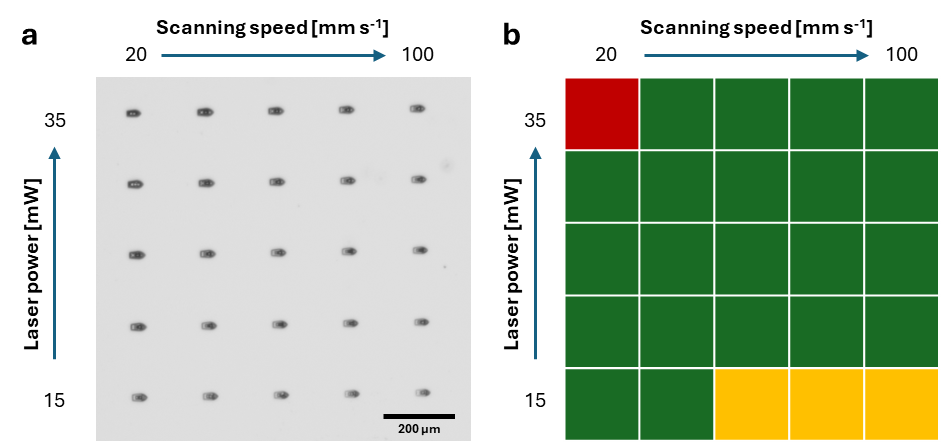


**Figure S19:** Printability window of DEI1 for varying printing parameters. **a)** 3D printed benchmark boat structures (50 µm × 25 µm × 40 µm) and **b)** evaluation of printing parameters.


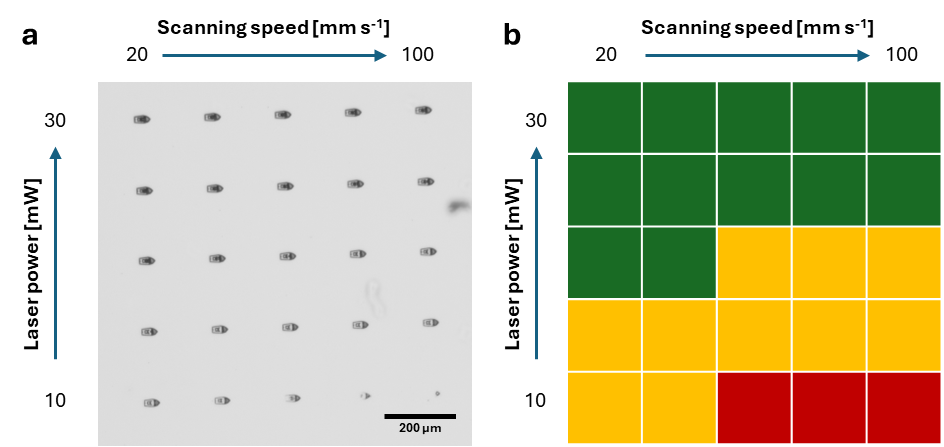


**Figure S20:** Printability window of DEI2 for varying printing parameters. **a)** 3D printed benchmark boat structures (50 µm × 25 µm × 40 µm) and **b)** evaluation of printing parameters.


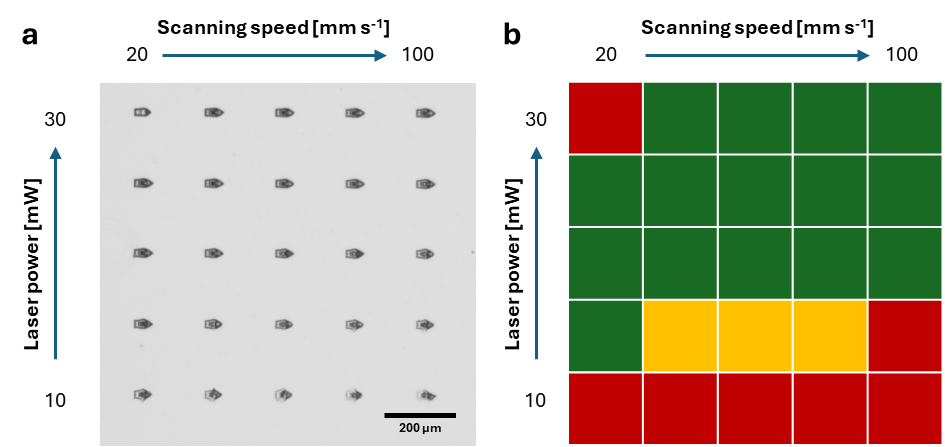


**Figure S21:** Printability window of DEI3 for varying printing parameters. **a)** 3D printed benchmark boat structures (50 µm × 25 µm × 40 µm) and **b)** evaluation of printing parameters.

Printability windows of highly sensitive reference systems (IP-S, PETA)

After printing the optimized DEIs, we compared the printing parameters to well-known frequently employed highly sensitive inks for MPLP. For this purpose, we selected IP-S (Nanoscribe GmbH) and a custom-made ink composed of 99.5 wt% PETA and 0.5 wt% DETC. Both materials show similar printability windows compared to the DEIs.


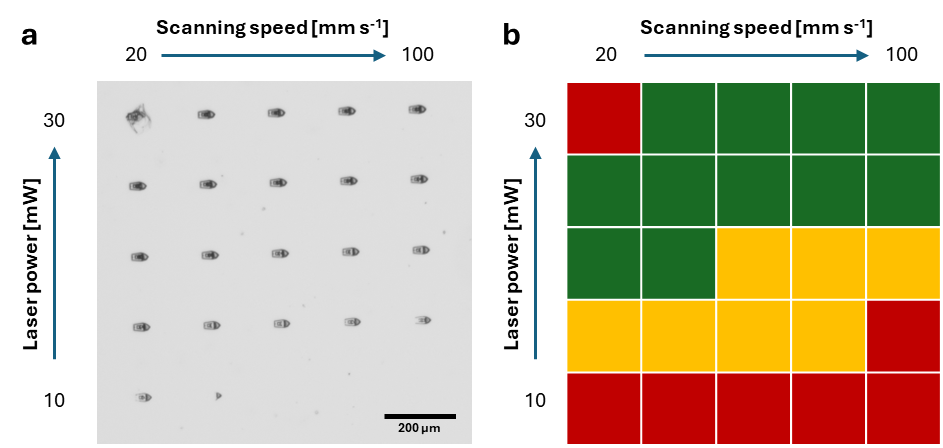


**Figure S22:** Printability window of IP-L (Nanoscribe GmbH) for varying printing parameters. **a)** 3D printed benchmark boat structures (50 µm × 25 µm × 40 µm) and **b)** evaluation of printing parameters.


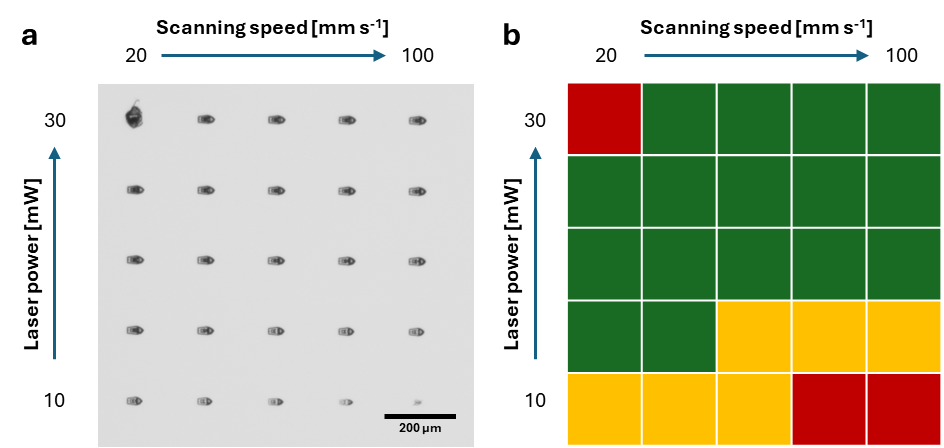


**Figure S23:** Printability window of PETA with 0.5 wt% DETC for varying printing parameters. **a)** 3D printed benchmark boat structures (50 µm × 25 µm × 40 µm) and **b)** evaluation of printing parameters.

Printability of optimized DEI and zinc-free comparative ink

In order to study the effect of zinc chloride on the printability of the ink, we first prepared a reference ink (RI1) without zinc chloride with similar mol% of acrylic monomers, covalent crosslinker PEGDA, and photoinitiator BAPO compared to DEI1 (see **Table S1** for DEI1 and **Table S2** for RI1).

**Table S1:** Ink composition of DEI1 with 5 wt% PEGDA.

| Compound | wt% | M (g mol^-1^) | mol% | AAc-mol% |
| --- | --- | --- | --- | --- |
| ZnCl_2_-AAc_2_ | 91.5 | 280.4 | 48.8% | 97.7% |
| AAc | 0 | 72.1 | 0% | 0% |
| PEGDA | 5 | 700.0 | 1.1% | - |
| BAPO | 3.5 | 418.5 | 1.3% | - |

**Table S2:** Ink composition of DEI1 with 5 wt% PEGDA without zinc chloride (RI1).

| Compound | wt% | M (g mol^-1^) | mol% | AAc-mol% |
| --- | --- | --- | --- | --- |
| ZnCl_2_-AAc_2_ | 0 | 280.4 | 0% | 0% |
| AAc | 84.7 | 72.1 | 97.7% | 97.7% |
| PEGDA | 9 | 700.0 | 1.1% | - |
| BAPO | 6.3 | 418.5 | 1.3% | - |

Next, the performance in MPLP of RI1 was compared to the performance of DEI1 (**Figure S24**). Printing of RI1 was not possible for the same broad range of printing parameters as for DEI1.


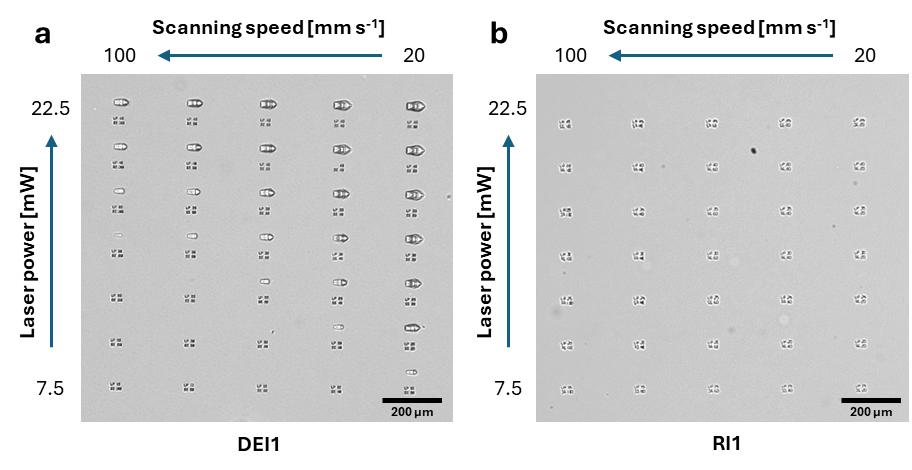


**Figure S24:** Printability windows of DEI1 (**a**) and RI1 (**b**) for printed boat structures (50 µm × 25 µm × 40 µm) with varying printing parameters. The ink formulations of both inks are summarized in **Table S1** for (**a**) and **Table S2** for (**b**).

By increasing the laser power, a very small printability window was found for MPLP. However, the 3D printed benchmark boat microstructures with these printing parameters were mechanically unstable (**Figure S25**).


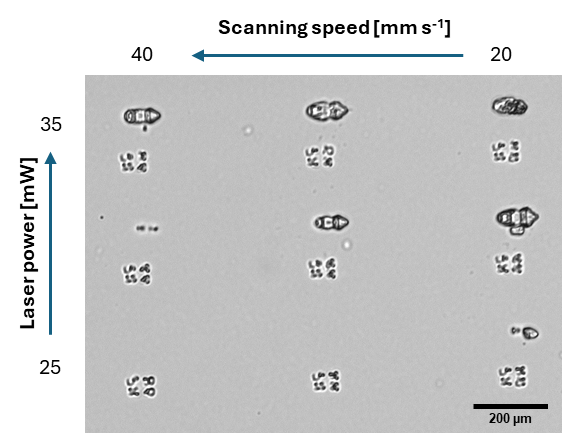


**Figure S25:** Printability window of RI1 (see also **b** in **Figure S24**) for printed boat microstructures (50 µm × 25 µm × 40 µm) with varying printing parameters and higher exposure doses. The ink formulation is summarized in **Table S2**.

Printability window of reference soft material ink (IP-PDMS)

After comparing the printability to highly sensitive inks, we compared the printing parameters also to the well-known frequently employed soft material ink IP-PDMS (Nanoscribe GmbH) for MPLP. Printing of the prepared DEIs was possible with a lower laser power compared to IP-PDMS.


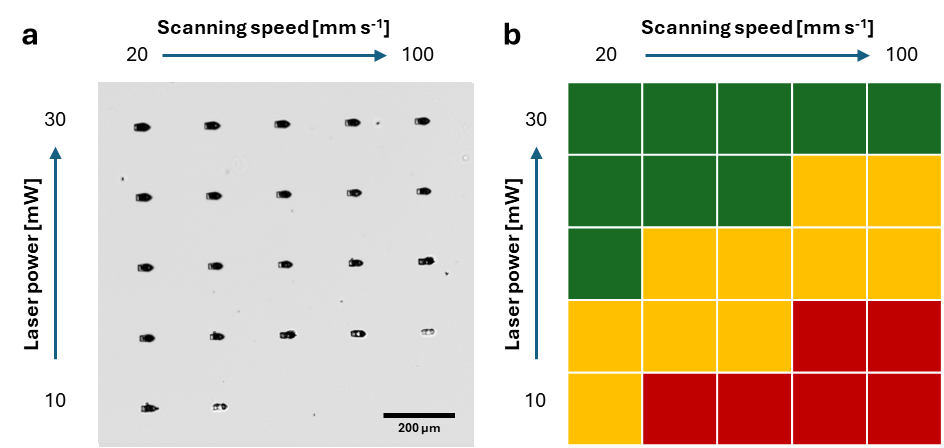


**Figure S26:** Printability window of IP-PDMS (Nanoscribe GmbH) for printed boat microstructures (50 µm × 25 µm × 40 µm) with varying printing parameters. **a)** 3D printed benchmark boat structures and **b)** evaluation of printing parameters.

Resolution tests

Lateral resolution was measured from printing single lines with varying distance from 0.4 µm and 1.6 µm as the distance at which the lines appear separated. To determine the highest possible resolution with the available setup, MPLP was performed using a 63× objective lens (NA = 1.4). The analysis was performed with optical microscopy and confocal fluorescence microscopy, respectively, and compared with the results obtained for IP-PDMS. We observed that the resolution in the dry and wet state was similar or even slightly better compared to that of the commercially available soft material ink IP-PDMS from Nanoscribe GmbH (**Table S3**).

**Table S3:** Achieved lateral resolution of the three selected main DEI systems in single line printing tests measured in the dry state from optical microscopy images and in the wet state from confocal fluorescence.

| Compound | Resolution (dry state) | Resolution (wet state) |
| --- | --- | --- |
| DEI1 | 1.0 µm | 1.0 µm |
| DEI2 | 1.0 µm | 1.0 µm |
| DEI3 | 1.0 µm | 1.2 µm |
| IP-PDMS | 1.2 µm | 1.2 µm |

The printed single lines for each material in **Table S3** are shown in **Figure S27**, **Figure S28**, **Figure S29**, and **Figure S30**.


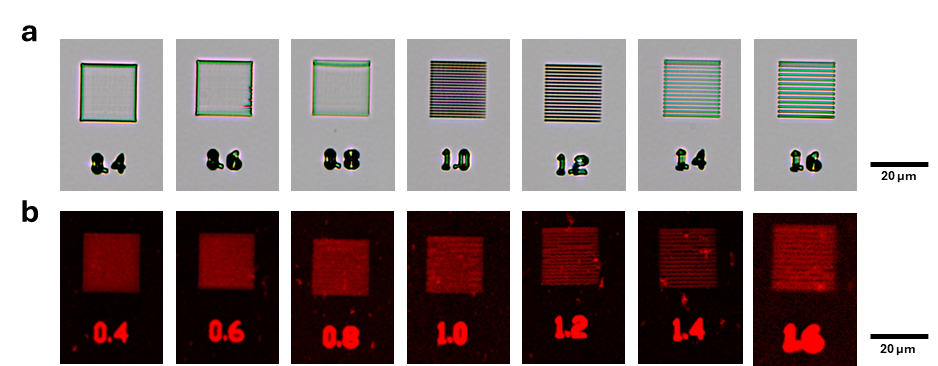


**Figure S27:** DEI1 single lines microprinted with a laser power of 5 mW and 0.1 mm s^-1^ focus velocity, and varying line pitch from 0.4 µm to 1.6 µm. **a)** Optical microscopy images in the dry state. **b)** Confocal fluorescence microscopy images in water using an excitation wavelength of 561 nm. The confocal images were recorded from rhodamine-B-methacrylate-incorporated structures.


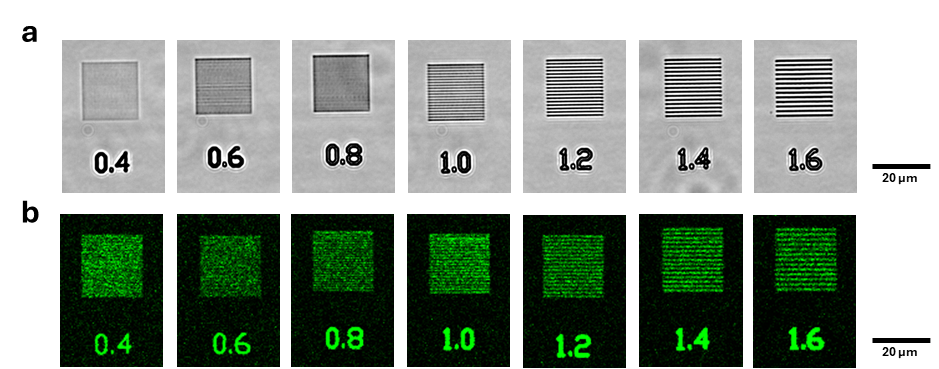


**Figure S28:** DEI2 single lines microprinted with a laser power of 5 mW and 0.1 mm s^-1^ focus velocity, and varying line pitch from 0.4 µm to 1.6 µm. **a)** Optical microscopy images in the dry state. **b)** Confocal fluorescence microscopy images in water using an excitation wavelength of 488 nm. The confocal images were recorded by using the autofluorescence of printed DEI2.


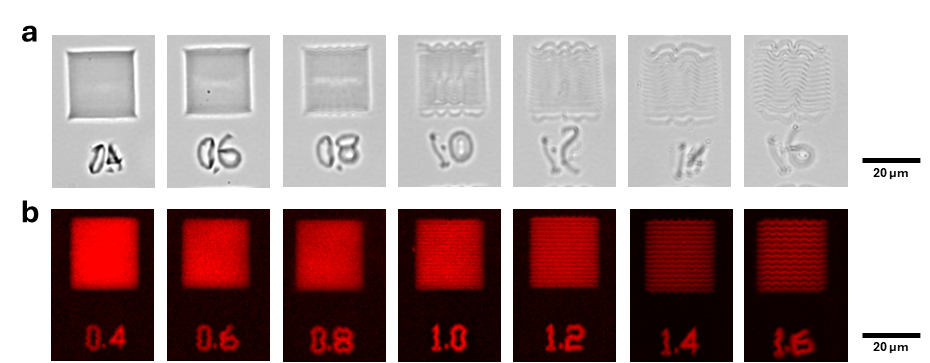


**Figure S29:** DEI3 single lines microprinted with a laser power of 15 mW and 0.1 mm s^-1^ focus velocity, and varying line pitch from 0.4 µm to 1.6 µm. **a)** Optical microscopy images in the dry state. **b)** Confocal fluorescence microscopy images in water using an excitation wavelength of 561 nm. The confocal images were recorded from rhodamine-B-methacrylate-incorporated structures.


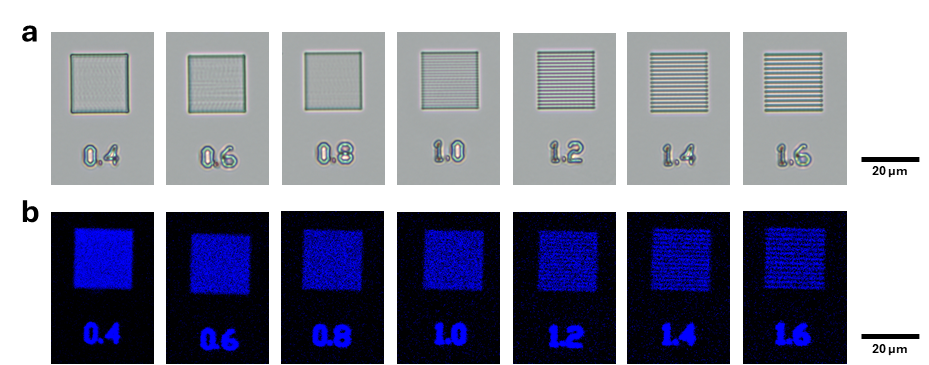


**Figure S30:** IP-PDMS single lines microprinted with a laser power of 5 mW and 0.1 mm s^-1^ focus velocity, and varying line pitch from 0.4 µm to 1.6 µm. **a)** Optical microscopy images in the dry state. **b)** Confocal fluorescence microscopy images in water using an excitation wavelength of 404 nm. The confocal images were recorded by using the autofluorescence of printed IP-PDMS.

Shape fidelity of printed structures in water

To show the achievable shape fidelity of DEI1, DEI2, and DEI3 in water, we printed hollow cylinders (outer and inner diameter 25 µm and 20 µm, respectively) with an enclosing net microstructure and performed confocal microscopy imaging of structures in water (**Figure S31**). The net microstructure consisted of 1 µm bars in the *xy*-plane with 2 µm × 2 µm windows.


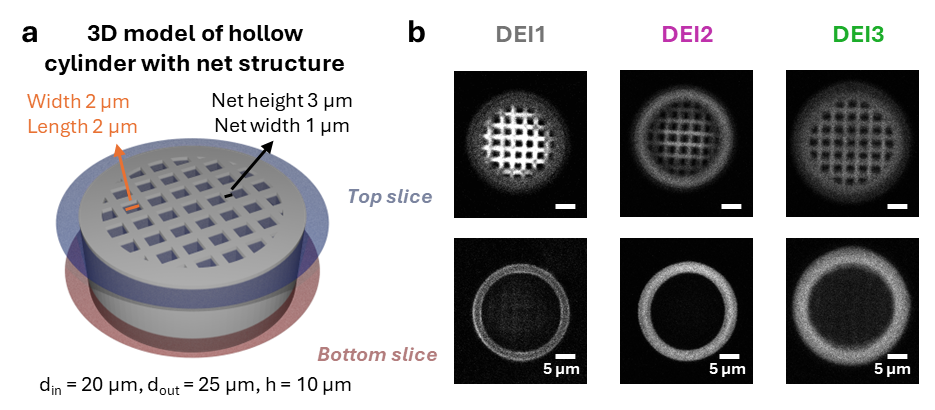


**Figure S31:** 3D printed net microstructure in water. **a)** 3D model of investigated hollow cylinder with net microstructure. The designed model was printed using the oil immersion mode with the 63× objective lens, a laser power of 30 mW, focus velocity of 10 mm s^-1^, slicing of 0.3 µm, and hatching of 0.2 µm.  **b)** Top and bottom slices of recorded confocal fluorescence microscopy images of the printed microstructures for DEI1 (with 0.01 wt% rhodamine B methacrylate) at 561 nm, DEI2 at 488 nm, and DEI3 at 488 nm.

To quantify the changes of the net microstructures in water to the 3D model, we used the top slice of the recorded stacks to measure the net widths and window withs (see **Table S4**). For this purpose, we measured the distances of 10 net widths and 10 window widths and averaged the outcome, respectively. DEI1 and DEI2 resembled with net widths of 0.97 µm and 1.00 µm the initially printed dimensions of 1.00 µm. DEI3 as the softest hydrophilic material in water with a net width of 1.14 µm showed minor relative deviations from the model dimensions due to swelling.

**Table S4:** Dimensions of the 3D printed net microstructures in water. The dimensions, *i.e.*, net and window width, were measured and analyzed from recorded confocal microscopy images at 10 different positions.

| Net microstructure | Net width (µm) | Window width (µm) |
| --- | --- | --- |
| Model | 1.00 | 2.00 |
| DEI1 | 0.97 ± 0.07 | 1.97 ± 0.13 |
| DEI2 | 1.00 ± 0.09 | 2.06 ± 0.11 |
| DEI3 | 1.14 ± 0.09 | 2.19 ± 0.09 |

Optical microscopy image of printed DEI1

The 3D printed ‘Eschenheimer Turm’ structure was imaged using optical microscopy in side view prior to sputter coating and SEM imaging (**Figure 3**). The flag on top of the tower collapsed during handling of dry samples.


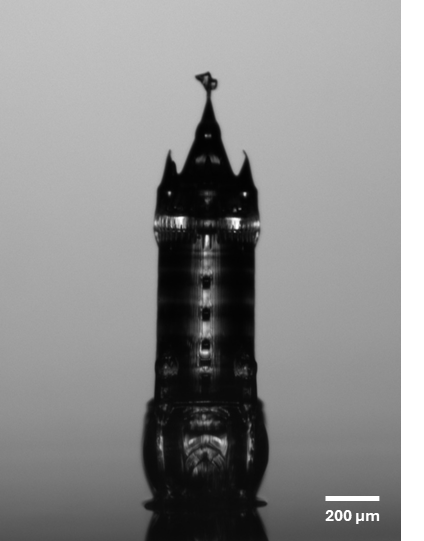


**Figure S32:** Optical microscopy image (side view) of DEI1 3D printed medieval tower ‘Eschenheimer Turm’ before sputter coating.

Printing parameters for the shown buildings and statues

The building and statue 3D structures were obtained from thingiverse.com and used without modifications. All stl files were shared under common license allowing its free use and sharing for non-commercial purposes.

For DEI1, the ‘Eschenheimer Turm’ (https://www.thingiverse.com/thing:4855222; BY-NC 4.0), ‘Atomium’ (https://www.thingiverse.com/thing:112476; CC BY-NC-SA 4.0) and the modeled elastic spring were printed with 100% laser power and 20 mm s^-1^ scanning speed, respectively.

The ‘Eiffel tower’ (https://www.thingiverse.com/thing:2239567; CC BY-ND 4.0) was printed with DEI2 using 100% laser power and 20 mm s^-1^ scanning speed.

‘Michelangelo’s David’ (https://www.thingiverse.com/thing:502967; CC BY-SA 3.0) was printed with DEI3 using 50% laser power and 40 mm s^-1^ scanning speed.

The ‘Al Wasl Dome’ (https://www.thingiverse.com/thing:6605755; CC BY-SA 3.0) was printed with DEI1+NIPAAm using 100% laser power and 10 mm s^-1^ scanning speed.

For DEI2+AAm, the ‘Statue of liberty’ (https://www.thingiverse.com/thing:4056644; CC BY 4.0), ‘Atomium’ (https://www.thingiverse.com/thing:112476; CC BY-NC-SA 4.0), and ‘Space space needle’ (https://www.thingiverse.com/thing:930296; CC BY-SA 3.0) were printed using 100% laser power and 20 mm s^-1^ scanning speed. The ‘Albero della vita’ (https://www.thingiverse.com/thing:922169; CC BY-SA 3.0) was printed using 100% laser power and 50 mm s^-1^ scanning speed.

FTIR spectroscopy of 3D printed DEI1

To study the chemical composition of the printed network, FTIR spectroscopy was performed on dry 3D printed DEI cubes (50 µm × 50 µm × 20 µm) using an FTIR microscope. 3D printed DEI1 was composed mainly of a very low crosslinked polyethylene glycol-crosslinked network with large amounts of AAc (**Figure S33**). The recorded spectra were similar to recorded FT-IR spectra of pure polyacrylic acid (PAA). In detail, the recorded FTIR spectra showed a large absorption band above ν = 3000 cm^-1^ showing the expected presence of free O-H groups. Compared to the IR spectra of ZnCl_2_-AAc_2_ (**Figure S14**), the C=O carboxyl band showed a hypsochromic shift back to higher wavenumbers (ν = 1698 cm^-1^) and appeared very sharp resembling the one of pure acrylic acid. As expected from the very low amount of employed covalent crosslinker PEGDA-M_n_700, the FTIR spectra showed only very minor unreacted acrylate C=C double bond signals around ν = 1560 cm^-1^ (see **Figure S33** and **Figure S14**).


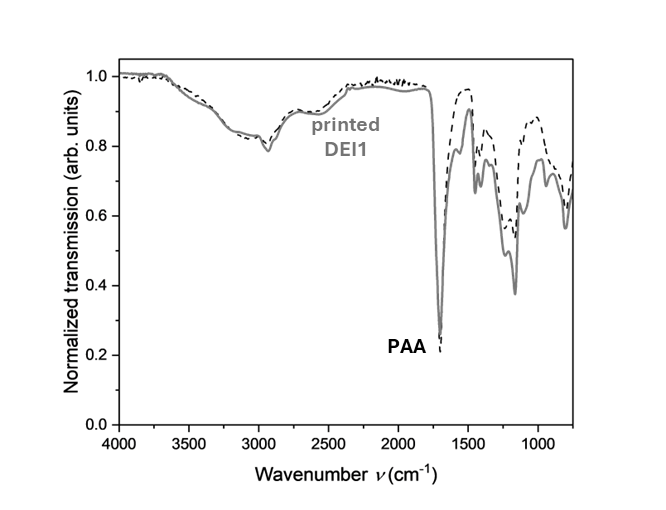


**Figure S33:** Recorded FTIR spectra of 3D printed DEI1 (50 µm × 50 µm × 20 µm) and polyacrylic acid (M_n_ 20000).

The recorded FTIR spectra of printed DEI2 and DEI3 follow the previously described trends for DEI1 such as a low content of present acrylates or hypsochromic shift (**Figure S34**).


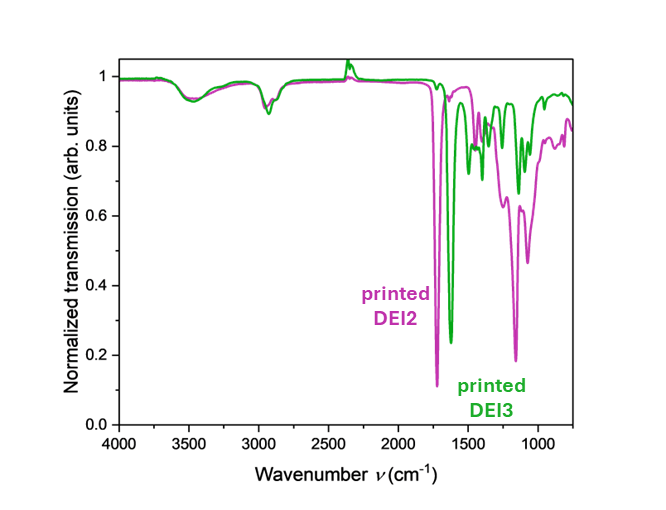


**Figure S34:** Recorded FTIR spectra of 3D printed cubes (50 µm × 50 µm × 20 µm) of DEI2 and DEI3.

Analysis of residual zinc ions

In addition to FTIR spectroscopy, we performed a highly sensitive UV-vis spectroscopic analysis using a zinc-binding organic dye (**Figure S35**) to investigate the chemical composition of the printed structures in-depth. To see if zinc ions are trapped and present in the DEI1 3D printed structures, we 3D printed millimeter-sized blocks with dimensions of 500 µm × 500 µm × 1000 µm and performed UV-vis spectroscopic analysis using zincon as a zinc-specific binding organic dye. After getting in contact with the 3D structures, the phosphate buffered zincon solution (188 µM) instantaneously showed a color change from orange to purple indicating the presence of zinc ions. The color change of the solution was observed in the recorded UV-vis spectra by a new band at 620 nm (**Figure S35**). A similar color change was detected after zincon solution got in contact with the ink DEI1. The observed color change and appearance of a new band at 620 nm align well with the complexation of zinc ions with zincon.^[2]^


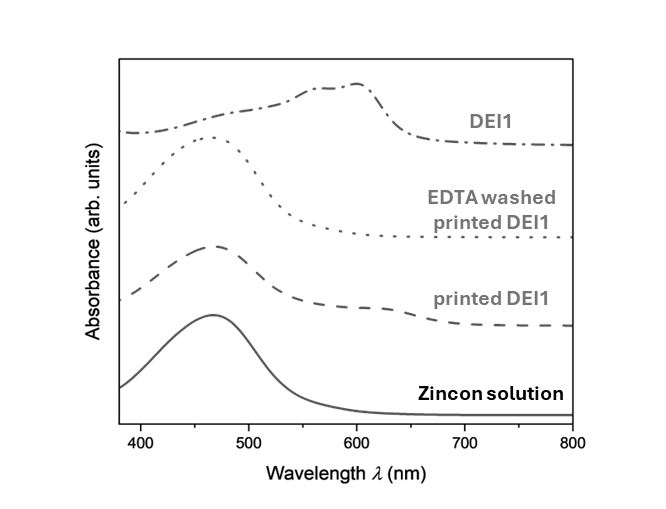


**Figure S35:** UV-vis spetra of zincon solution before and after contact with a 3D printed DEI1 structure, EDTA washed 3D printed DEI1 structure, and the DEI1 ink. The stack is shown with an offset. The additional EDTA wash does not lead to the color change of the zincon (analyte) solution indicating the absence of zinc ions.

Removing residual zinc ions was possible by performing an additional washing step with an aqueous solution with a chelat binding ligand, i.e. saturated aqueous ethylenediaminetetraacetic acid (EDTA) solution, which was expected to strongly bind to the transition metal. The additional development step removed residual zinc ions entirely from the printed PEG-crosslinked acrylic acid network as shown by UV spectroscopic analysis. In detail, the zincon solution exhibited the same absorption behavior before and after contact to the EDTA washed DEI1 structure (**Figure S35**).

In addition to the spectroscopic analysis, we performed thermogravimetric analysis (TGA) of the 3D printed blocks with and without EDTA washing to study the thermal stability and amount of residue (**Figure S36**). Thermogravimetric analysis of the printed structures with and without residual zinc ions showed similar weight residues after heating to 600 °C indicating that the amount of zinc ions in the structures still present in the structures is very low (**Figure S36**).


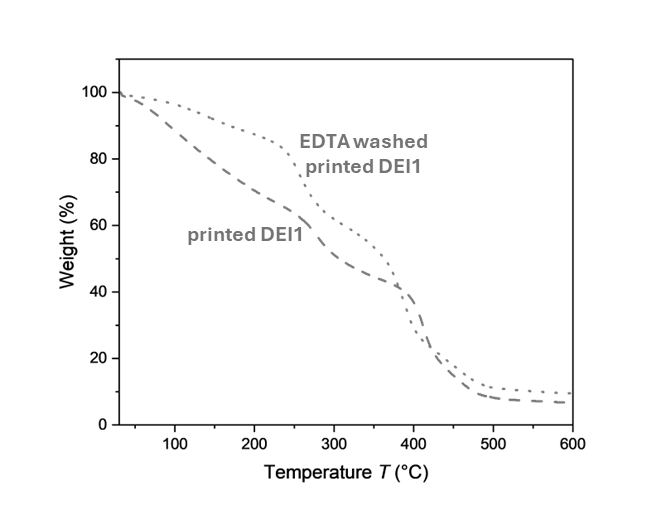


**Figure S36:** Thermogravimetric analysis of 3D printed DEI1 structures.

Furthermore, the printed structures with residual zinc showed a higher affinity to binding water from the atmosphere and a slightly increased thermal stability toward decomposition.

Mechanical characterization of printed DEIs


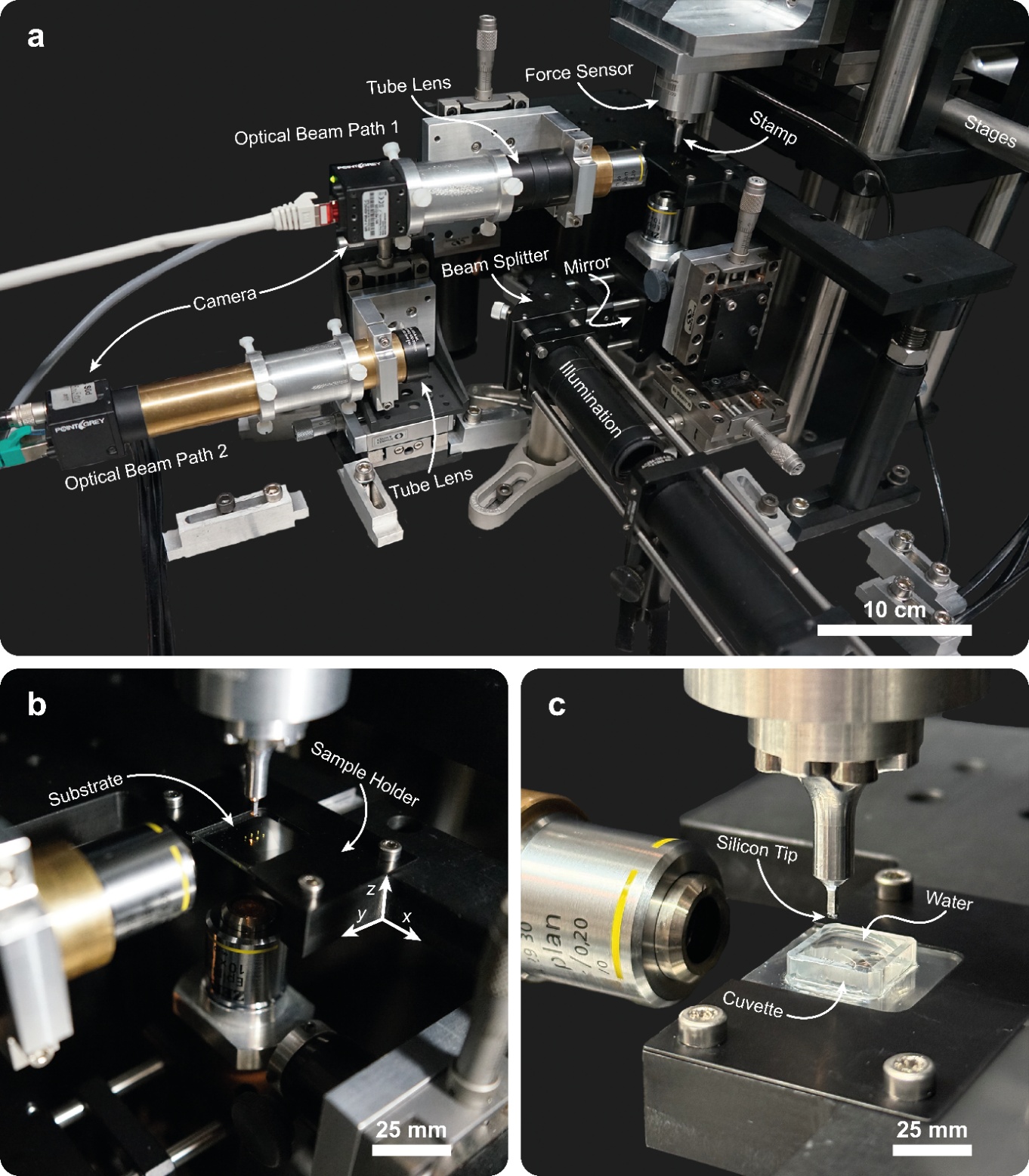


**Figure S37: a)** Overview of the home-built setup for uniaxial-loading experiments and mechanical characterization, illustrating the main parts of the setup. Bottom illumination of the substrate is included in optical beam path 2; back illumination and light sources are not depicted. **b)** Sample configuration for measurements in air, the substrate is fixed in the sample holder and placed underneath the custom-made stamp with silicon tip, in front of the two objective lenses. One pillar of the sample array is illuminated by the second optical-beam path. **c)** Sample configuration for measurements in aqueous media, the cuvette functions as sealed reservoir for deionized water during the measurements. Mounting of the substrate is the same as in **a**, displayed materials are DEI2+AAm (**a**) and DEI3 (**b**).

The main parts of the setup included two optical microscopes (optical beam path 1 and 2, ZEISS Epiplan 10×, NA = 0.2) with attached cameras (Point Grey Research, Blackfly BFLY-PGE-50H5C) for imaging the sample from the bottom and side during the experiment, a force sensor (ME-Meßsysteme, K3D35 500 mN) with a custom-made aluminum stamp (cross-section 1 mm^2^), attached to automated translation stages (TRA25CC, Newport), and a sample holder. To provide a smoother surface of the stamp, a piece of silicon of the same cross-section was added to the tip. Illumination was provided by back illumination (Schott, KL 2500 LED) and from the bottom via optical beam path 2 (Schott, KL 1500 LCD), which is not depicted in **Figure S37A.** The setup was placed on an air-cushion optical table (RS 2000, Newport).

Measurements of dry samples were performed directly using the setup. For measuring the samples in water, a cuvette was glued to the glass slide. The cuvette functions as a reservoir to store and keep the samples in aqueous media. Both sample configurations are depicted in **Figure S37B** and **Figure S37C**.

The recorded force is a function of the prescribed displacement (**Figure S39**), the effective spring constant is obtained from the slope of a linear regression model fitted to the linear regime of the loading cycles. By calculating the Young’s modulus from the effective spring constant, the change of the sample geometry of the different materials, through shrinkage after development or swelling in water, is included by taking pictures of the samples in the experimental setup before conducting the experiments and analyzing them with the software ‘ImageJ’ to measure the height and diameter of the samples. The Young’s modulus for each sample is averaged over all loading cycles for each prescribed geometry corrected strain, and the modulus for each material is averaged over all measured samples. Geometry parameters and results of the Young’s moduli are listed in **Table S5** and **Table S6**, respectively. Data analysis programmed in MATLAB, optical micrographs of the samples and supporting videos can be found in the data repository published with this work (DOI:10.11588/DATA/UP26J7).

**Table S5:** Results of mechanical characterization of 3D printed DEIs in air.

| Printed  Material | Height  *h* (µm) | Diameter  *d* (µm) | Strain  linear regime | Corrected strain linear regime | Young’s moduli in air (MPa) |
| --- | --- | --- | --- | --- | --- |
| HEA-pure | 216.8 ± 6.0 | 291.6 ± 2.9 | 4% | 5.5% | 30.276 ± 0.068 |
| DEI1 | 283.4 ± 4.8 | 309.6 ± 6.1 | 10% | 10.6% | 36.344 ± 0.070 |
| DEI2 | 300.9 ± 2.8 | 386.5 ± 4.1 | 10% | 10.0% | 12.606 ± 0.046 |
| DEI3 | 264.8 ± 4.0 | 367.8 ± 2.8 | 6% | 6.8% | 30.193 ± 0.043 |
| DEI1+NIPAAm | 276.0 ± 4.1 | 346.1 ± 2.7 | 6% | 10.9% | 35.897 ± 0.044 |
| DEI2+AAm | 294.4 ± 3.4 | 384.0 ± 3.2 | 10% | 6.1% | 14.364 ± 0.040 |

**Table S6:** Results of mechanical characterization of 3D printed DEIs in water.

| Printed  Material | Height  *h* (µm) | Diameter  *d* (µm) | Strain  linear regime | Corrected strain linear regime | Young’s moduli in water (MPa) |
| --- | --- | --- | --- | --- | --- |
| HEA-pure | 252.0 ± 6.1 | 321.9 ± 3.3 | 4% | 4.7% | 5.679 ± 0.064 |
| DEI1 | 390.5 ± 5.1 | 499.1 ± 6.1 | 10% | 7.7% | 0.575 ± 0.056 |
| DEI2 | 297.6 ± 6.5 | 387.5 ± 1.2 | 10% | 10.1% | 10.526 ± 0.045 |
| DEI3 | 463.8 ± 5.0 | 641.5 ± 2.5 | 6% | 3.9% | 0.265 ± 0.027 |
| DEI1+NIPAAm | 400.2 ± 5.8 | 490.0 ± 3.7 | 6% | 7.5% | 0.393 ± 0.042 |
| DEI2+AAm | 303.5 ± 6.8 | 404.7 ± 2.9 | 10% | 5.9% | 9.867 ± 0.053 |

The temporal measurement protocol for the first force loading and unloading cycle of the DEI2 measurement in air is depicted in **Figure S38** as an example. The time between the loading and unloading was 1.6 s for all measurements. The delay between the 10 cycles was set to 40 s.


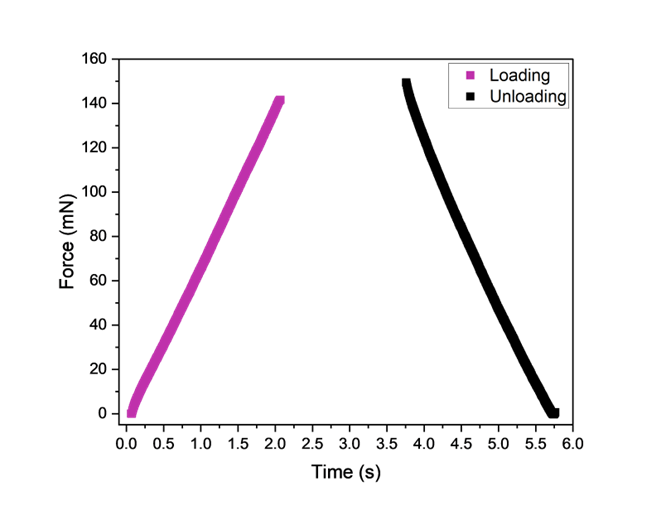


**Figure S38:** Selected data of first force loading and unloading cycle *versus* time for printed DEI2 cylinder in air.

The measured force-strain curves are depicted for one measured pillar of the six different materials, respectively. The corrected strain includes the geometry measurement obtained by ‘ImageJ’ for the Young’s modulus calculation.


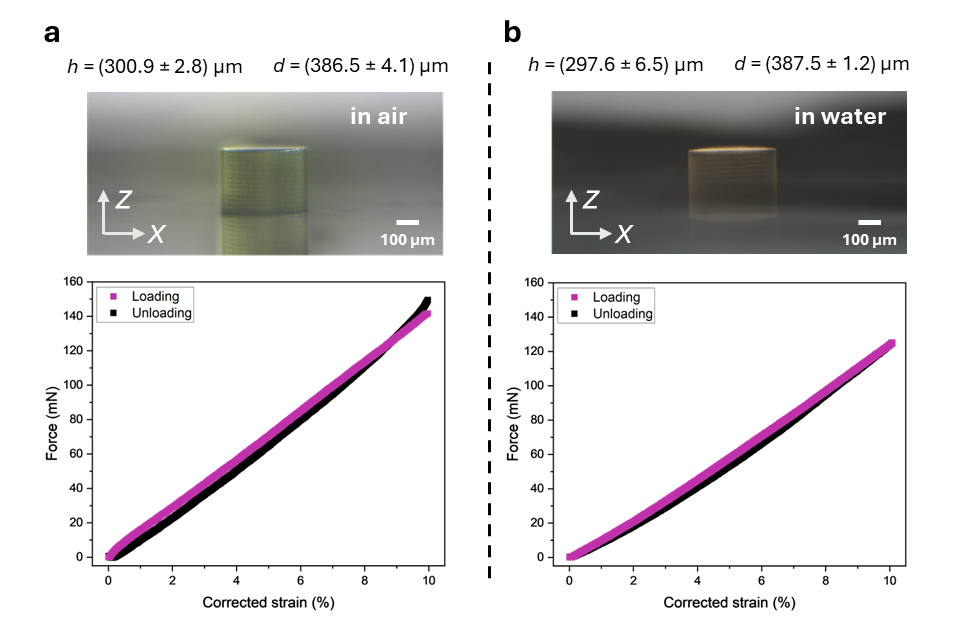


**Figure S39:** One measured force-strain loading and unloading cycle of one printed DEI2 cylinder in air (**a**) and in water (**b**).


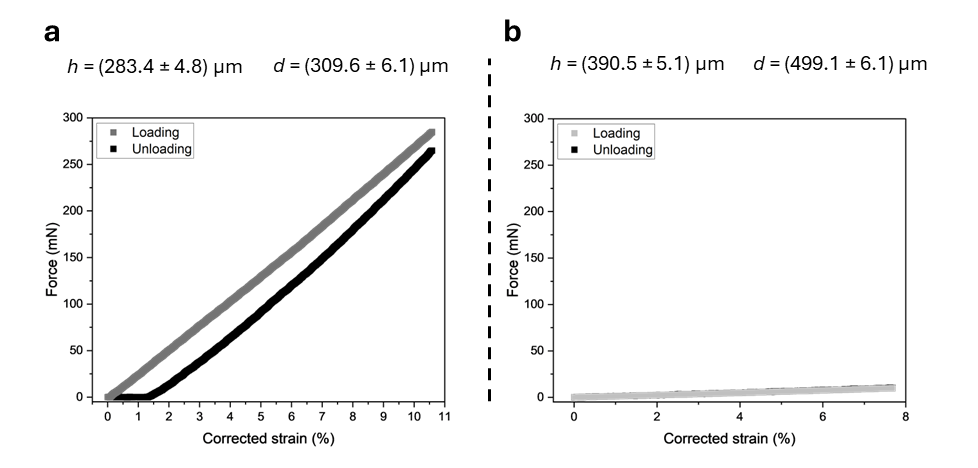


**Figure S40:** One measured force-strain loading and unloading cycle of one printed DEI1 cylinder in air (**a**) and in water (**b**).


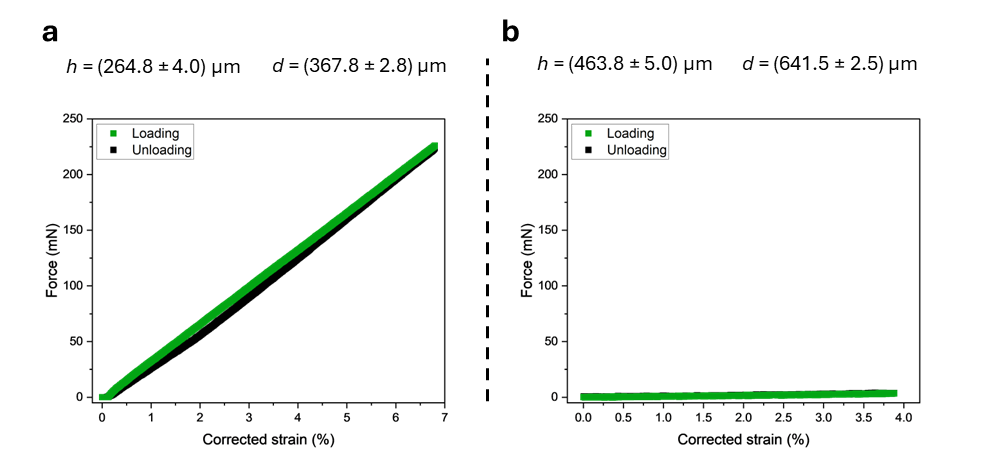


**Figure S41:** One measured force-strain loading and unloading cycle of one printed DEI3 cylinder in air (**a**) and in water (**b**).


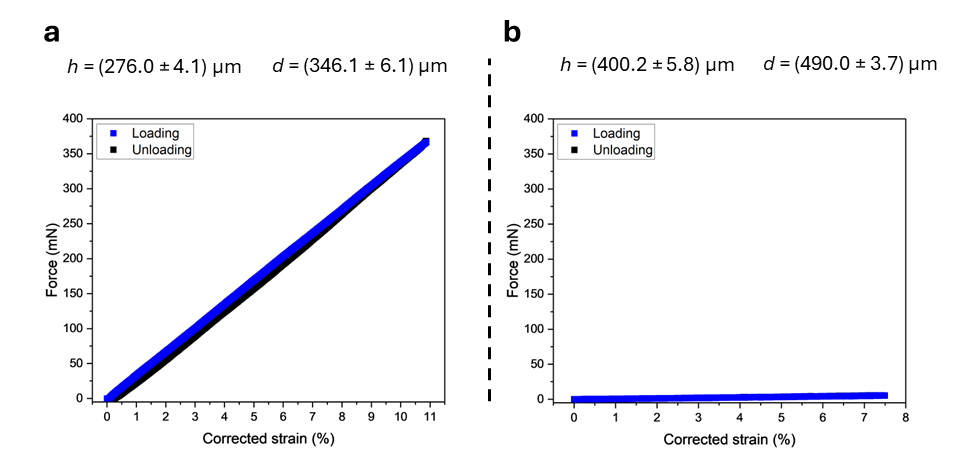


**Figure S42:** One measured force-strain loading and unloading cycle of one printed DEI1+NIPAAm cylinder in air (**a**) and in water (**b**).


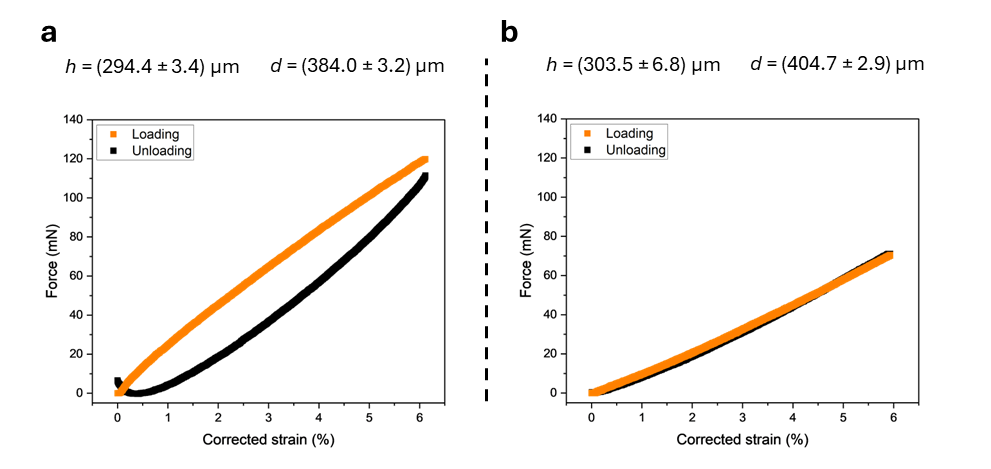


**Figure S43:** One measured force-strain loading and unloading cycle of one printed DEI2+AAm cylinder in air (**a**) and in water (**b**).


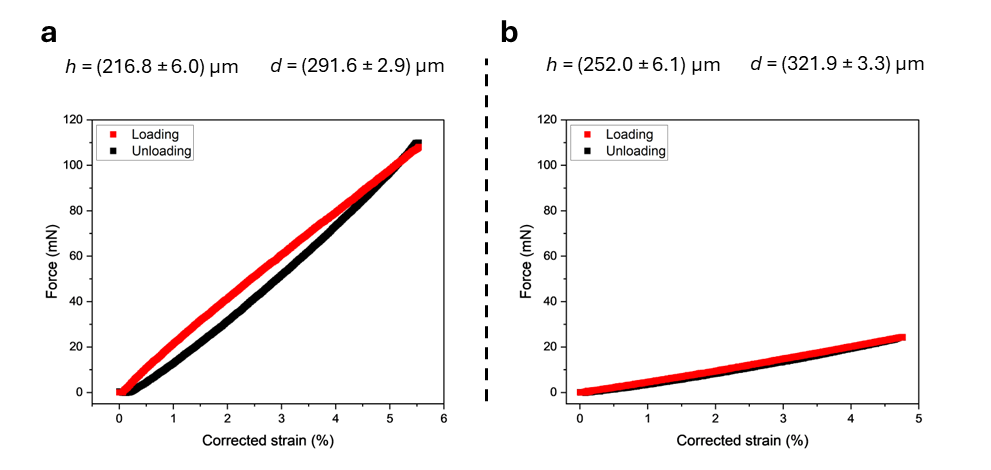


**Figure S44:** One measured force-strain loading and unloading cycle of one printed HEA-pure cylinder in air (**a**) and in water (**b**).

The Young’s moduli of the printed HEA-pure, DEI1+NIPAAm, and DEI2+AAm are shown in **Figure S45**.


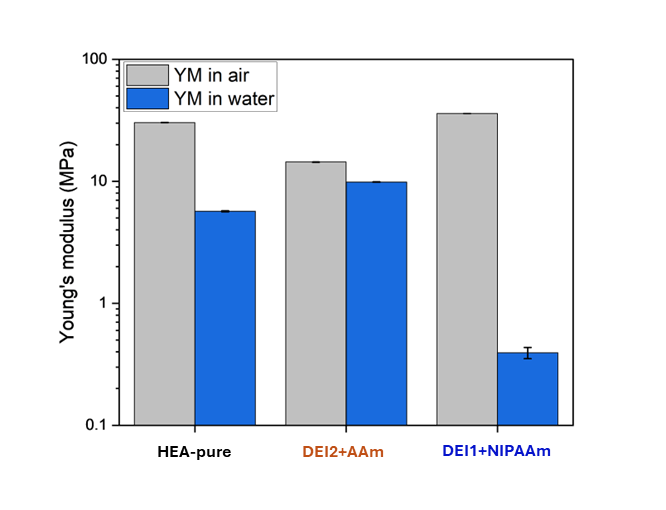


**Figure S45:** Young’s moduli of HEA-pure, DEI2+AAm, and DEI1+NIPAAm, and IP-PDMS. The Young’s moduli were calculated from force-strain displacement curves in the linear regime (see **Table S5** and **Table S6**).

To compare the compression measurements with a reference system, we have selected IP-PDMS (Nanoscribe GmbH) as a commercially available reference system. First, we verified the reproducibility of measured Young’s moduli of our setup. For this purpose, we printed a similar cubic structure with dimensions of 100 µm × 100 µm × 50 µm using the 25× objective, slicing and hatching 0.3 µm, laser power 50 mW, and scanning speed 80 mm s^-1^ as reported by Nanoscribe GmbH in their nanoindentation experiment. The sample was analyzed using the compression setup and showed Young’s moduli of (15.290 ± 0.911) MPa. This value very much resembles the reported 15.3 MPa by Nanoscribe GmbH. ^[3]^


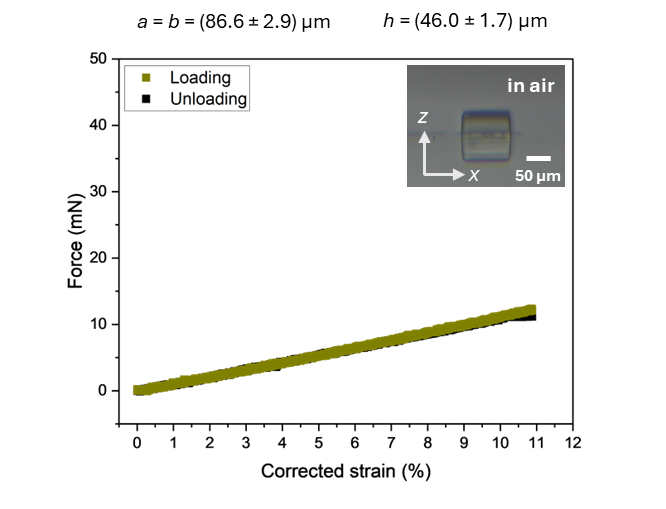


**Figure S46:** One measured force-strain loading and unloading cycle of one 3D printed IP-PDMS cube with dimensions of 100 µm × 100 µm × 50 µm in air as a reference measurement.

As a next step, we printed a cylinder of IP-PDMS of the same geometry as previously employed for the DEIs (**Figure S47**). To avoid heat accumulation, we reduced the laser power from 50 mW to 40 mW to use the same scanning speed of 80 mm s^-1^. The Young’s modulus of the printed cylinder was found to be (5.975 ± 0.204) MPa underlining the strong influence of the printing parameters on the resulting mechanical properties as it has been described previously for other inks. ^[4]^


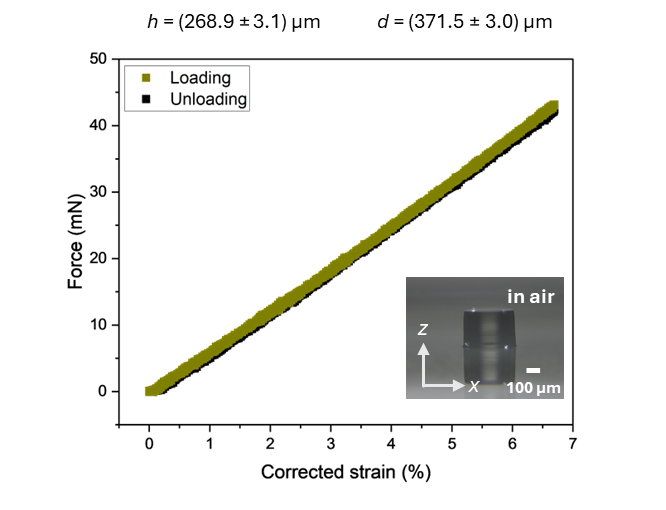


**Figure S47:** One measured force-strain loading and unloading cycle of the 3D printed IP-PDMS cylinder in air printed with the 25× objective. The cylinder (300 µm height, 400 µm diameter) has been printed with 80 mm s^-1^, 40 mW, 0.3 µm slicing, and 0.3 µm hatching.

To finally compare the mechanical properties using similar printing conditions as the DEI, we printed and analyzed four cylinders of IP-PDMS with the 10× objective (with foil) ^[5]^, same geometry, and printing parameters as we previously employed for the DEIs (**Figure S48**). We observed a severe shrinkage of around 30% and 20% (pillars in air and in water) which was larger compared to that the shrinkage observed for the printed DEIs. The smaller printability window and severe shrinkage suggest that the used printing parameters do not offer relevant quality in structure fabrication. This was further supported by compression measurements of the printed IP-PDMS pillars yielding around (0.452 ± 0.121) MPa in air and (0.489 ± 0.068) MPa in water. This result highly deviated from the measured and reported Young’s moduli 15.3 MPa by the vendor (Nanoscribe GmbH). ^[3]^ Thus, our results support the recommendation of Nanoscribe to use IP-PDMS with the 25× objective.


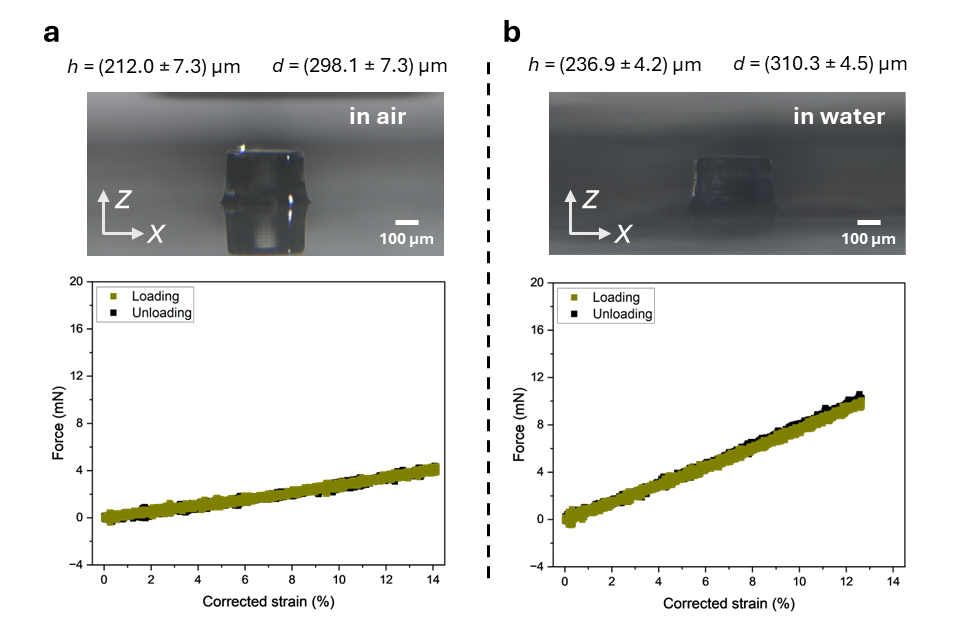


**Figure S48:** One measured force-strain loading and unloading cycle of one 3D printed IP-PDMS cylinder in air (**a**) and in water (**b**). The cylinder (300 µm height, 400 µm diameter) has been printed using the 10× objective and similar printing conditions as for the DEIs. The IP-PDMS cylinder shows severe shrinkage (20-30%) compared to the reference sample printed with the 25× objective (**Figure S47**).

The performed reference measurements using IP-PDMS (Nanoscribe GmbH) are summarized in **Table S7** and **Table S8**.

**Table S7:** Results of IP-PDMS reference measurements in compression experiments in air.

| Objective | Geometry | Height  *h* (µm) | Strain  linear regime | Corrected strain linear regime | Young’s moduli in air (MPa) |
| --- | --- | --- | --- | --- | --- |
| 25× | Rectangular | 46.0 ± 1.7 | 10% | 10.9% | 15.290 ± 0.911 |
| 25× | Cylindrical | 268.9 ± 3.1 | 6% | 6.7% | 5.975 ± 0.204 |
| 10× | Cylindrical | 212.0 ± 7.3 | 10% | 14.2% | 0.452 ± 0.121 |

**Table S8:** Results of IP-PDMS reference measurements in compression experiments in water.

| Objective | Geometry | Height  *h* (µm) | Strain  linear regime | Corrected strain linear regime | Young’s moduli in water (MPa) |
| --- | --- | --- | --- | --- | --- |
| 10× | Cylindrical | 236.9 ± 4.2 | 10% | 12.7% | 0.489 ± 0.068 |

Comparison of mechanical properties with available inks for dip-in mode MPLP

**Table S9:** Mechanical properties of reported inks available for MPLP preparation of larger structures in dip-in mode (measured * - dry and in ° - aqueous media). ^[6-8]^

| Inks for MPLP of larger structures using the dip-in mode | Young’s modulus  (MPa) |
| --- | --- |
| Nanoscribe IP-Q* | 3100 |
| Nanoscribe IP-S* | 2100 |
| DEGRAD INX© X100* | 50 – 60 |
| Nanoscribe IP-PDMS* | 15.3 |
| HYDROTECH INX© X200° | 3 – 4 |
| **This work – DEIs*** | **12** – **36** |
| **This work - DEIs°** | **0.26** – **10** |

Extending functionality by mixing DEIs with N-isopropyl acrylamide

After printing a DEI with acrylamide, we selected *N*-isopropyl acrylamide (NIPAAm) as another monomer with responsive properties. To achieve multiresponsive behavior towards multiple stimuli such as temperature, pH, and calcium, ^[9,10]^ we aimed for combining NIPAAm with the previously identified DES ZnCl_2_-AAc_2_. Similar to acrylamide in ZnCl_2_-HEA_2_, NIPAAm, added in a 1:2 weight ratio, readily dissolved in the ZnCl_2_-AAc_2_ DES. It is worth noting that NIPAAm showed no lower critical solubility transition (LCST) in the mixture when heated to 50 °C (**Figure S49**), drastically extending its processing window.


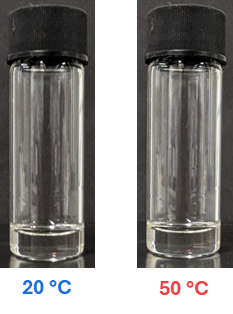


**Figure S49:** Dissolved *N*-isopropyl acrylamide (33 wt%) in ZnCl_2_-AAc_2_ (67 wt%) at room temperature and 50 °C.

The NIPAAm mixed with ZnCl_2_-AAc_2_ was used to prepare an ink (DEI1+NIPAAm) together with PEGDA as crosslinker and BAPO as photoinitiator (**Table S10**).

**Table S10:** Ink composition of DEI1+NIPAAm with *N*-isopropyl acrylamide.

| Compound | wt% | M (g mol^-1^) | mol% |
| --- | --- | --- | --- |
| ZnCl_2_-AAc_2_ | 61.0 | 280.4 | 43.3% |
| NIPAAm | 30.5 | 113.2 | 53.6% |
| PEGDA | 5 | 700.0 | 1.4% |
| BAPO | 3.5 | 418.5 | 1.7% |

DEI1+NIPAAm exhibited an excellent printability window as the other DEIs (**Figure S50**).


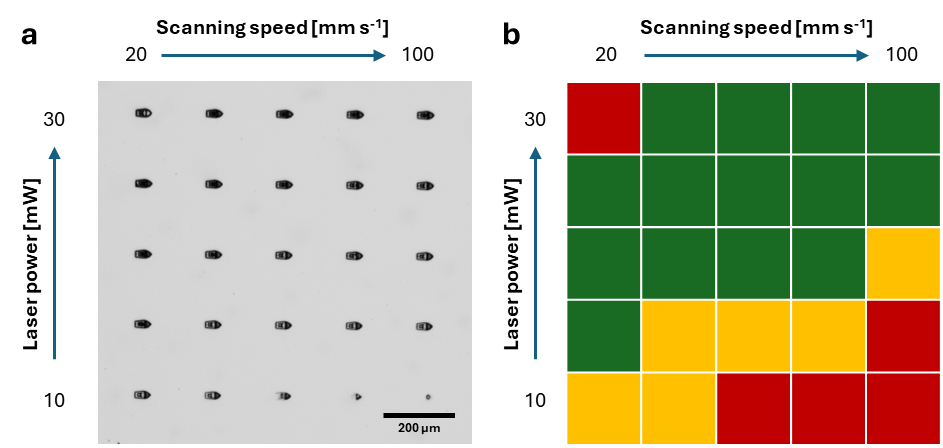


**Figure S50:** Printability window of DEI1+NIPAAm with *N*-isopropyl acrylamide. **a)** 3D printed benchmark boat structures (50 µm × 25 µm × 40 µm) and **b)** evaluation of the printing parameters.

3D printed temperature-, pH-, and calcium-responsive DEI1+NIPAAm

We 3D printed micrometric buckyballs with DEI1+NIPAAm in dimensions of 100 µm × 100 µm × 100 µm and recorded the printed structures upon heating and cooling between room temperature and 60 °C in water (**Video S5**).


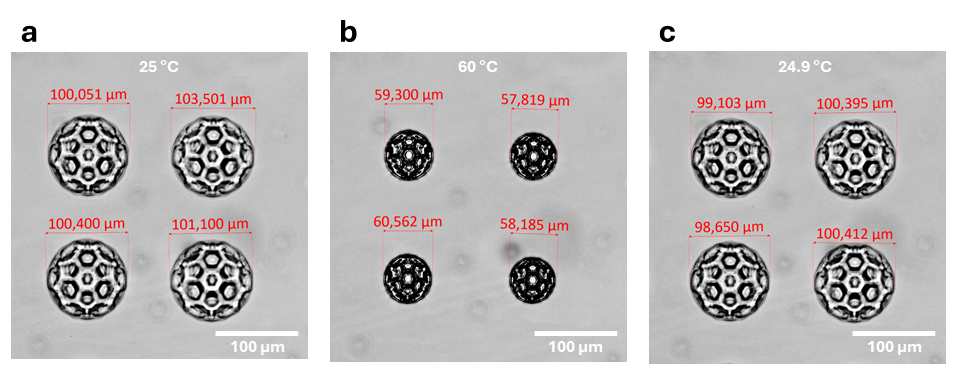


**Figure S51:** Temperature response of 3D printed buckyball (54 µm × 54 µm × 50 µm, laser power 20 mW, and scanning speed 40 mm s^-1^) of DEI1+NIPAAm upon heating to 60 °C. The buckyballs shrink from a swelling factor of S_V_ = 7 (in **a**) to about S_V_ = 1.3 (in **b**) in volume. The dimensions recover upon cooling the microstructures back to room temperature (in **c**).

Upon heating, we observed large shrinkage of the buckyballs with a change in swelling factor from S_V_ = 7 at 25 °C to S_V_ = 1.0 at 60 °C with respect to the printed dimensions (**Figure S51**). This shrinkage was recovered when the buckyballs were cooled back to room temperature.

The microprinted structures also exhibited the expected swelling or shrinkage upon addition of basic aqueous solution or calcium ions, respectively (see **Figure S52** and **Figure S53**).


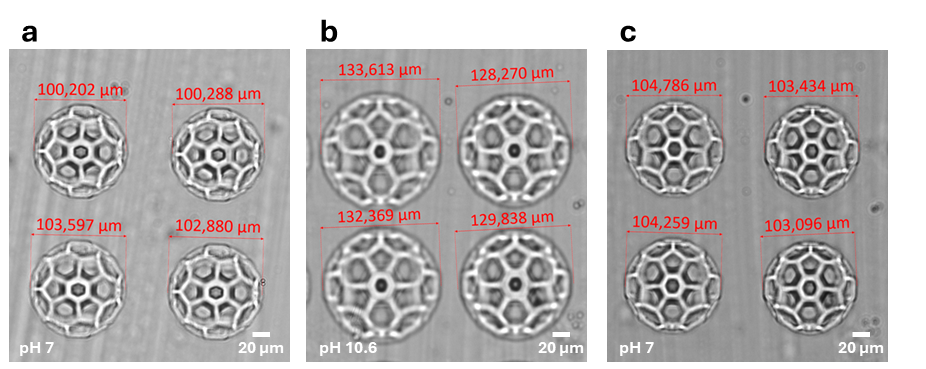


**Figure S52:** pH response of 3D printed buckyball (54 µm × 54 µm × 50 µm, laser power 20 mW, and scanning speed 40 mm s^-1^) of DEI1+NIPAAm between pH = 7 and pH = 10.6. The buckyballs swell by a swelling factor of S_V_ = 7 (in **a**) to about S_V_ = 14.7 (in **b**) in volume. The dimensions recover upon bringing the microstructures back to pH = 7 (in **c**).


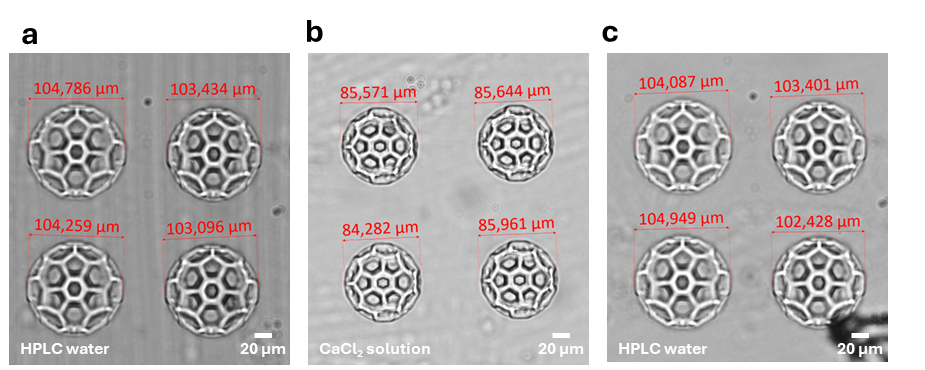


**Figure S53:** Calcium response of 3D printed buckyball (54 µm × 54 µm × 50 µm, laser power 20 mW, and scanning speed 40 mm s^-1^) of DEI1+NIPAAm between HPLC water and aqueous calcium chloride solution (0.24 g mL^-1^). The buckyballs shrink from a swelling factor of S_V_ = 7 (in **a**) to about S_V_ = 4 (in **b**) in volume. The dimensions recover upon washing the microstructures with HPCL water (in **c**).

To demonstrate the potential of combining different DEIs into multimaterial structures, we designed and printed gripper structures with dimensions of 620 µm × 620 µm × 240 µm. The structural base for a bilayered actuator was printed with DEI3. The temperature-responsive layer was printed with DEI1+NIPAAm on top of the DEI3 base structure. The printed structures presented temperature-induced bending of the gripper arms upon heating and cooling between room temperature and 60 °C (**Figure S54** and **Video S6**).


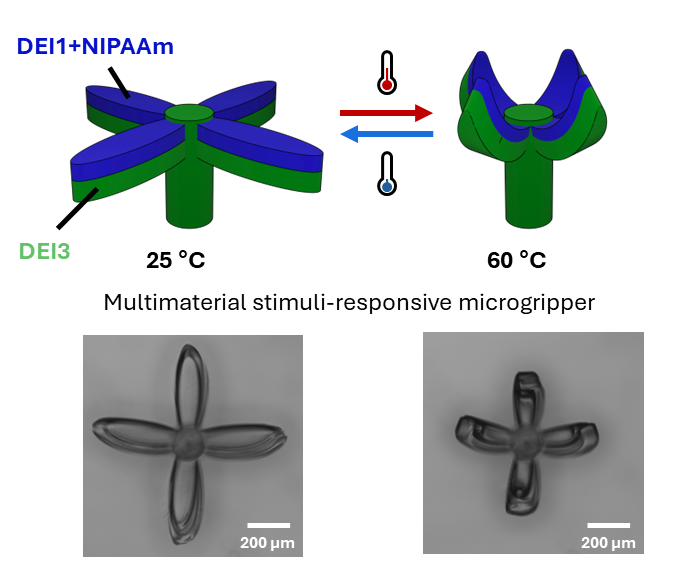


**Figure S54:** Temperature response of 3D printed multimaterial (DEI1+NIPAAm and DEI3) gripper (620 µm × 620 µm × 240 µm). Schematic representation (top) and optical microscopy images (bottom) of the observed temperature response.

Extending functionality by mixing DEIs with acrylamide

To show the versatility of DEIs, we mixed acrylamide, a monomer with a high melting point, in a 1:1 mass ratio with ZnCl_2_-HEA_2_. Since acrylamide was readily miscible in the DES, we prepared an ink (DEI2+AAm) by adding PEGDA and BDEABP (**Table** **S11**).

**Table S11:** Ink composition of DEI2+AAm with acrylamide.

| Compound | wt% | M (g mol^-1^) | mol% |
| --- | --- | --- | --- |
| ZnCl_2_-HEA_2_ | 47.5 | 368.5 | 16.0% |
| AAm | 47.5 | 71.1 | 83.9% |
| PEGDA | 4 | 700.0 | 0.7% |
| BDEABP | 1 | 324.5 | 0.4% |

Remarkably, DEI2+AAm showed excellent printability using various laser powers and scanning speeds similar to DEI2 (**Figure S55**).


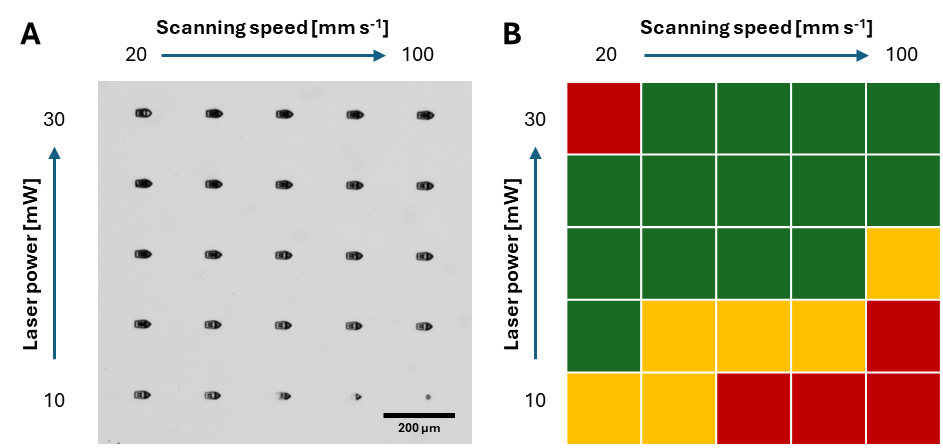


**Figure S55:** Printability window of DEI2+AAm for varying printing parameters. **a)** 3D printed benchmark boat structures (50 µm × 25 µm × 40 µm) and **b)** evaluation of printing parameters.

DEI2+AAm was used for MPLP of various structures such as a statue of liberty (2 mm height), space needle (1.5 mm height), ‘Atomium’ (600 µm), and ‘Albero della vita’ (600 µm).


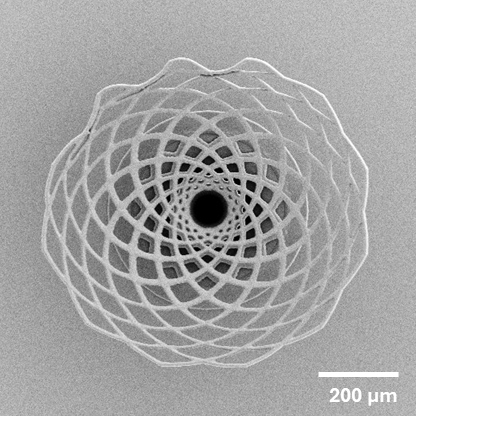


**Figure S56:** SEM image of DEI2+AAm 3D printed ‘Albero della vita’ in top view.


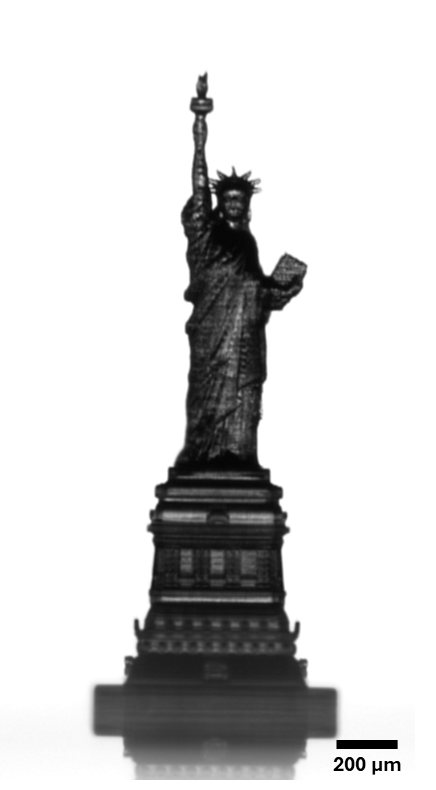


**Figure S57:** Optical microscopy image (side view) of DEI2+AAm printed statue of liberty before sputter coating.


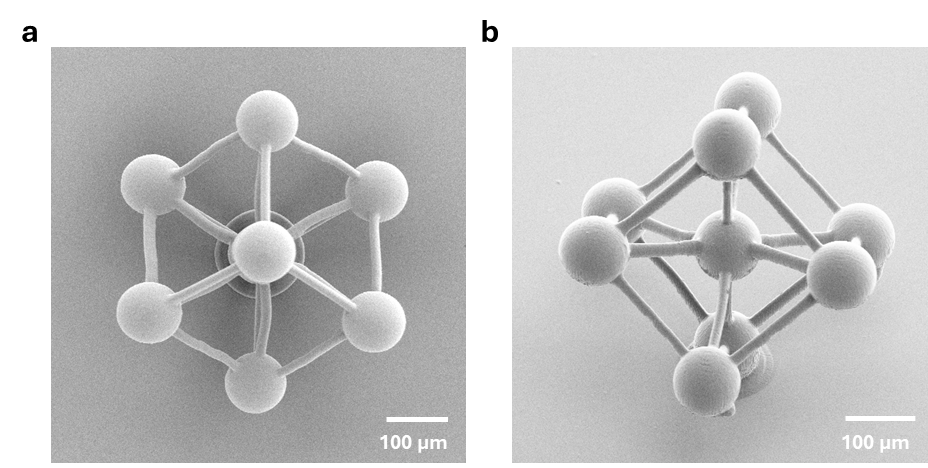


**Figure S58:** SEM images of DEI2+AAm 3D printed ‘Atomium’ **a)** in top view and **b)** in side view.

SEM images of 3D printed cubic grids

We have used DEI2 and DEI2+AAm for 3D printing cubic grids. The SEM images of the printed structures in **Figure 1** are shown from different angles in **Figure S59** and **Figure S60**.


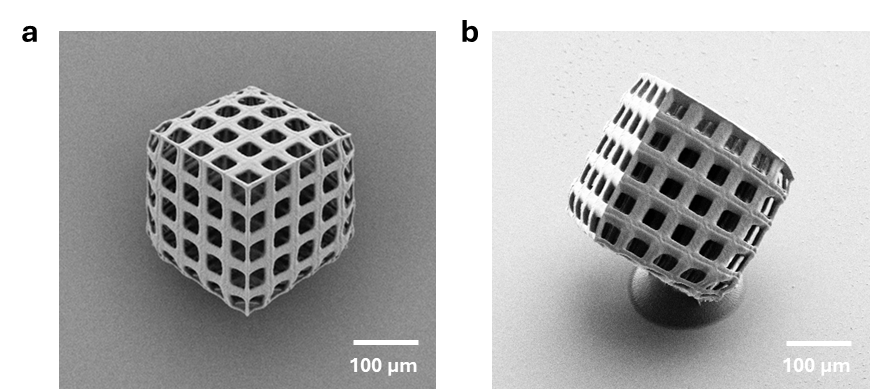


**Figure S59:** SEM images of DEI2 3D printed cubic grid **a)** in top view and **b)** in side view.


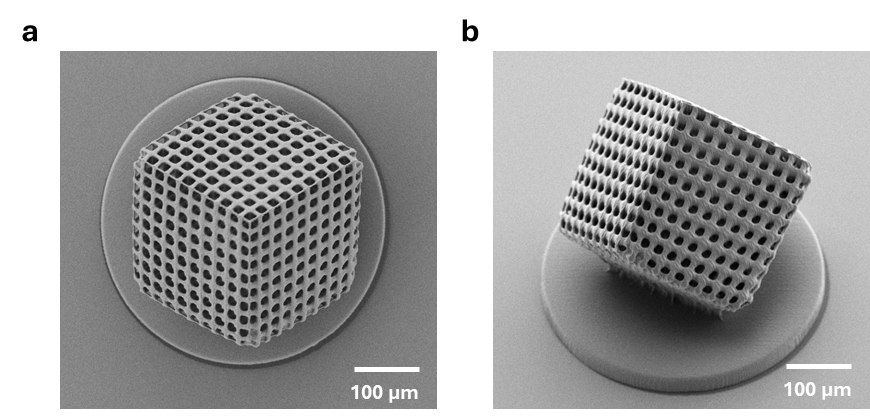


**Figure S60:** SEM images of DEI2+AAm 3D printed cubic grid **a)** in top view and **b)** in side view.

References

[1] M. Schmid, D. Ludescher, H. Giessen, *Opt. Mater. Express* **2019**, *9*, 4564.

[2] A. Kocyła, A. Pomorski, A. Krężel, *J. Inorg. Biochem.* **2017**, *176*, 53.

[3] Nanoscribe GmbH & Co. KG, Nanoguide, *IP-PDMS photoresin*, **April 2025**, https://support.nanoscribe.com/hc/en-gb/articles/360022218600-IP-PDMS.

[4] Q. Hu, G. A. Rance, G. F. Trindade, D. Pervan, L. Jiang, A. Foerster, L. Turyanska, C. Tuck, D. J. Irvine, R. Hague, R. D. Wildman, *Addit. Manuf.* **2022**, *51*, 102575.

[5] A. Toulouse, S. Thiele, K. Hirzel, M. Schmid, K. Weber, M. Zyrianova, H. Giessen, A. M. Herkommer, M. Heymann, *Opt. Mater. Express* **2022**, *12*, 3801.

[6] Nanoscribe GmbH & Co. KG, Products, A*vailable photoresins for dip-in MPLP*, **June 2025**, https://www.nanoscribe.com/fileadmin/Nanoscribe/PDF/Product_Folder/Folder-Printing-Materials-2025.pdf.

[7] BioINX, Products, DEGRAD *photoresin for dip-in MPLP*, **June 2025**, https://bioinx.com/sites/default/files/product_files/Degrad%20INX%20X100%20Product%20Information%20Sheet.pdf

[8] BioINX, Products, HYDROTECH *photoresin for dip-in MPLP*, **June 2025**, https://bioinx.com/sites/default/files/product_files/hydrotech%20X200%20product%20information%20sheet.pdf

[9] P. Schattling, F. D. Jochum, P. Theato, *Polym. Chem.* **2014**, *5*, 25.

[10] Y.-W. Lee, H. Ceylan, I. C. Yasa, U. Kilic, M. Sitti, *ACS Appl. Mater. Interfaces* **2021**, *13*, 12759.
